# Preference uncertainty accounts for developmental effects on susceptibility to peer influence in adolescence

**DOI:** 10.1038/s41467-021-23671-2

**Published:** 2021-06-22

**Authors:** Andrea M. F. Reiter, Michael Moutoussis, Lucy Vanes, Rogier Kievit, Edward T. Bullmore, Ian M. Goodyer, Peter Fonagy, Peter B. Jones, NSPN Consortium, Edward Bullmore, Edward Bullmore, Raymond Dolan, Ian Goodyer, Peter Jones, Tobias Hauser, Sharon Neufeld, Rafael Romero-Garcia, Michelle St Clair, Petra Vértes, Kirstie Whitaker, Becky Inkster, Gita Prabhu, Cinly Ooi, Umar Toseeb, Barry Widmer, Junaid Bhatti, Laura Villis, Ayesha Alrumaithi, Sarah Birt, Aislinn Bowler, Kalia Cleridou, Hina Dadabhoy, Emma Davies, Ashlyn Firkins, Sian Granville, Elizabeth Harding, Alexandra Hopkins, Daniel Isaacs, Janchai King, Danae Kokorikou, Christina Maurice, Cleo McIntosh, Jessica Memarzia, Harriet Mills, Ciara O’Donnell, Sara Pantaleone, Jenny Scott, Matilde Vaghi, Anne-Laura van Harmelen, Andrea Reiter, Raymond J. Dolan

**Affiliations:** 1 Max Planck UCL Centre for Computational Psychiatry and Ageing Research, University College London, London, UK; 2 Wellcome Centre for Human Neuroimaging, University College London, London, UK; 3 Department of Child and Adolescent Psychiatry, Psychosomatics and Psychotherapy, University Hospital of Würzburg, Würzburg, Germany; 4 MRC Cognition and Brain Sciences Unit, Cambridge, UK; 5 Department of Psychiatry, University of Cambridge Clinical School, Cambridge, UK; 6 Department of Clinical, Educational and Health Psychology, University College London, London, UK; 7 University of Bath, Bath, UK; 8 Cambridge Centre for Ageing and Neuroscience (Cam-CAN), University of Cambridge, Cambridge, UK; 9 Faculty of Education, University of Cambridge, Cambridge, UK

**Keywords:** Cognitive neuroscience, Development of the nervous system, Learning and memory, Social behaviour, Social neuroscience

## Abstract

Adolescents are prone to social influence from peers, with implications for development, both adaptive and maladaptive. Here, using a computer-based paradigm, we replicate a cross-sectional effect of more susceptibility to peer influence in a large dataset of adolescents 14 to 24 years old. Crucially, we extend this finding by adopting a longitudinal perspective, showing that a within-person susceptibility to social influence decreases over a 1.5 year follow-up time period. Exploiting this longitudinal design, we show that susceptibility to social influences at baseline predicts an improvement in peer relations over the follow-up period. Using a Bayesian computational model, we demonstrate that in younger adolescents a greater tendency to adopt others’ preferences arises out of a higher uncertainty about their own preferences in the paradigmatic case of delay discounting (a phenomenon called ‘preference uncertainty’). This preference uncertainty decreases over time and, in turn, leads to a reduced susceptibility of one’s own behaviour to an influence from others. Neuro-developmentally, we show that a measure of myelination within medial prefrontal cortex, estimated at baseline, predicts a developmental decrease in preference uncertainty at follow-up. Thus, using computational and neural evidence, we reveal adaptive mechanisms underpinning susceptibility to social influence during adolescence.

## Abstract (web summary)

People often change their preferences to conform with others. Using a longitudinal design, the authors show that such conformity decreases over the course of adolescence and that this reduction in conformity is accompanied by a decreasing degree of uncertainty about what to like.

## Introduction

Across many species, adolescence is a key period for social development^1^. Animal and human studies suggest social interactions are more salient for adolescents than for adults^2^. Adolescence is also a period of enhanced susceptibility to peer influence^3–10^, an effect that remains highly relevant in the digital age, where adolescents are increasingly exposed to a range of social media^11^. Higher susceptibility to social influence is traditionally thought to have particular relevance for the emergence of psychopathology and health damaging real-life behaviours^12–17^. Thus, adolescents smoke and drink more alcohol when in the presence of peers, and peers’ substance consumption is a predictor of a teenager’s own substance use^15,18,19^. The prevalence of self-injury behaviours, as well as unprotected sexual intercourse, are often related to a social contagion effect during adolescence^20–22^. However, recent alternative accounts frame susceptibility to social influence during adolescence in a less maladaptive context^23,6,24–27^: social influence can change behaviour for the better, an effect widely used for adaptive ends in education and psychotherapy. Susceptibility to peer influence can be associated with higher psycho-social integration in young adolescents^5,23^. Thus, a higher tendency to integrate social influence into one’s own decisions might be an adaptive ingredient in healthy social development during adolescence^23^, a period of life characterised by a shift in social orientation away from the parents towards one’s peer group.

Although peer influences on decision-making during adolescence have been widely investigated, several important questions remain unanswered. First, claims on social susceptibility and its real-life consequences in adolescents mostly rely on cross-sectional designs and modest sample sizes. Here, we applied a longitudinal design in a large cohort of adolescents and young adults to study peer influence on a well characterised task assessing inter-temporal preferences. Inter-temporal (‘delay’) discounting is a key measure of temporal impulsivity, which has been shown repeatedly to decrease from childhood to young adulthood^28–30^. Longitudinal designs are important for addressing developmental questions as they provide a basis for disentangling cohort or sampling effects from developmental trajectories.

Second, though previous studies have established higher conformity towards peers in adolescents than in adults, it remains unclear why this is the case. The adult conformity literature suggests two distinct routes towards conformity, namely informational influences (‘copy-when-uncertain’, observing others to gain information regarding behaviours that are currently adaptive) and normative influences (adhering to social norms / expectations of the other, bringing direct benefits through belonging, social tension reduction and acceptance)^31,32^. Many findings on peer influence in adolescent psychology are implicitly interpreted within the framework of normative influence, suggesting the pursuit of social acceptance and sensitivity towards social evaluation by peers is a significant determinant of adolescent decision-making^1,3,12,33^. Here, we tested an alternative hypothesis, namely that informational influences underlie higher conformity in adolescence. To this end, we built on our previously validated (Bayesian) probabilistic reasoning model^34^ that describes conformity in terms of a learning effect. In brief, if people are uncertain about exactly what to like (‘preference’ or ‘taste’ uncertainty)^35^ they can learn about it by adopting the preferences of their peers.

Third, we were interested in the co-development of brain structures that are relevant for the expression of preference uncertainty. We previously found that social susceptibility during a delay discounting task is mediated by plasticity in the medial prefrontal cortex (mPFC)^36^. A key mechanism contributing to plasticity itself is myelination^37^, a process that extends throughout adolescence and into adulthood. Recent studies in humans have observed that individual differences in the unfolding of these myelin growth trajectories are sensitive to inter-individual differences, including the expression of psychopathology^38,39^, environmental effects^40^ and social cognition^41^, rendering myelin a candidate marker in the co-development of brain-behaviour relationships. Likewise, animal^42–45^ and human^41,46^ work has highlighted the importance of medial prefrontal myelin plasticity for socially relevant behaviour across development. This background provided a motivation to ask, using in-vivo myelin-sensitive magnetisation transfer MRI^39^, whether mPFC myelin is related to developmental effects on preference uncertainty, which in turn impacts on social susceptibility.

We show that social susceptibility, in terms of preference shifts in a delay discounting task, not only decreases with age cross-sectionally, but also longitudinally. Longitudinally, we demonstrate that susceptibility to peer influence in our experimental paradigm as measured at baseline predicts the quality of peer relationships over follow-up, which might point to an adaptive role of social susceptibility in healthy adolescents. Using our computational model, we show that such preference uncertainty, here in the paradigmatic case of delay discounting, decreases both with age and over the course of our longitudinal follow-up, and longitudinal change is strongest in the youngest of our sample. Crucially, both cross-sectional and longitudinal developmental effects on social susceptibility, in terms of preference shifts in our social delay discounting task, are accounted for by developmental changes in preference uncertainty, suggesting that higher preference uncertainty in younger adolescents is a key mechanism facilitating peer influence in teenagers. On the neural level, we demonstrate that a myelin marker in medial prefrontal cortex predicts a developmental decrease in preference uncertainty over our longitudinal follow-up period.

## Results

**Fig. 1:**
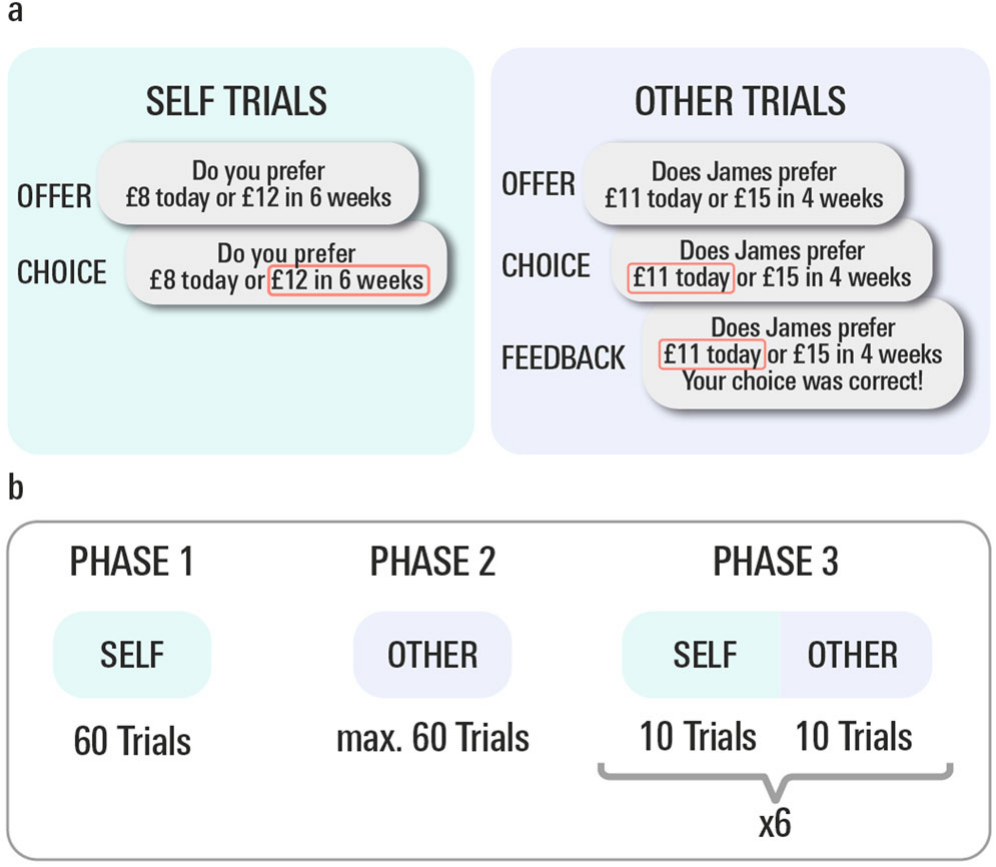
Social delay discounting task. **a** Example trial for ‘self’ and ‘other’ trial types. In self trials, participants see an offer of a smaller amount of money they can receive on the same day or a larger amount of money they can receive after a variable delay period. Subjects were instructed to indicate their preference according to their true personal preference and, to enforce expression of true preferences, they were told that one trial would be chosen at random to determine their pay out. In ‘other’ trials, subjects chose between the smaller, immediate and the larger, delayed option on behalf of another person, and received feedback on these choices thereby enabling them to learn the others’ delay discounting preferences. Individual scores of susceptibility to social influence were defined as the degree of change in one’s own discount rate towards the preference of a social partner (i.e., log *k*_self_phase3_ – log k_self_phase1_). **b** Experimental Design. In phase 1, participants completed 60 ‘self’ trials, in phase 2, participants engaged in ‘other’ trials for a minimum of 20 trials, until either the participant got eight correct answers out of the most recent 10 trials, or until 60 learning trials were completed.

To probe a susceptibility to social influence, we used a social version of a delay discounting task (Fig. 1). In short, this task allows us to measures a person’s temporal discounting coefficient (how much less a future reward is worth, depending on the delay of its delivery) as well as changes in their discount function pre vs. post learning about someone else’s discount preferences^34,36,47^ (See Fig. 1 and ‘Methods’ for details). Here, in line with previous accounts^34,48^, we defined susceptibility to social influence as the degree of change in one’s own discount rate towards that of a social partner, following exposure to the discounting preferences of a social partner *(log k*_self_phase3_ *– log* k_self_phase1_).

Analyses on subjects’ choice behaviour, reaction times, delay discounting preferences, as well as effects of the ability to learn from others, and the effect of the other’s delay discounting preferences are reported in the Supplementary Information.

### Cross-sectional and longitudinal developmental effects of susceptibility to social influence

**Fig. 2:**
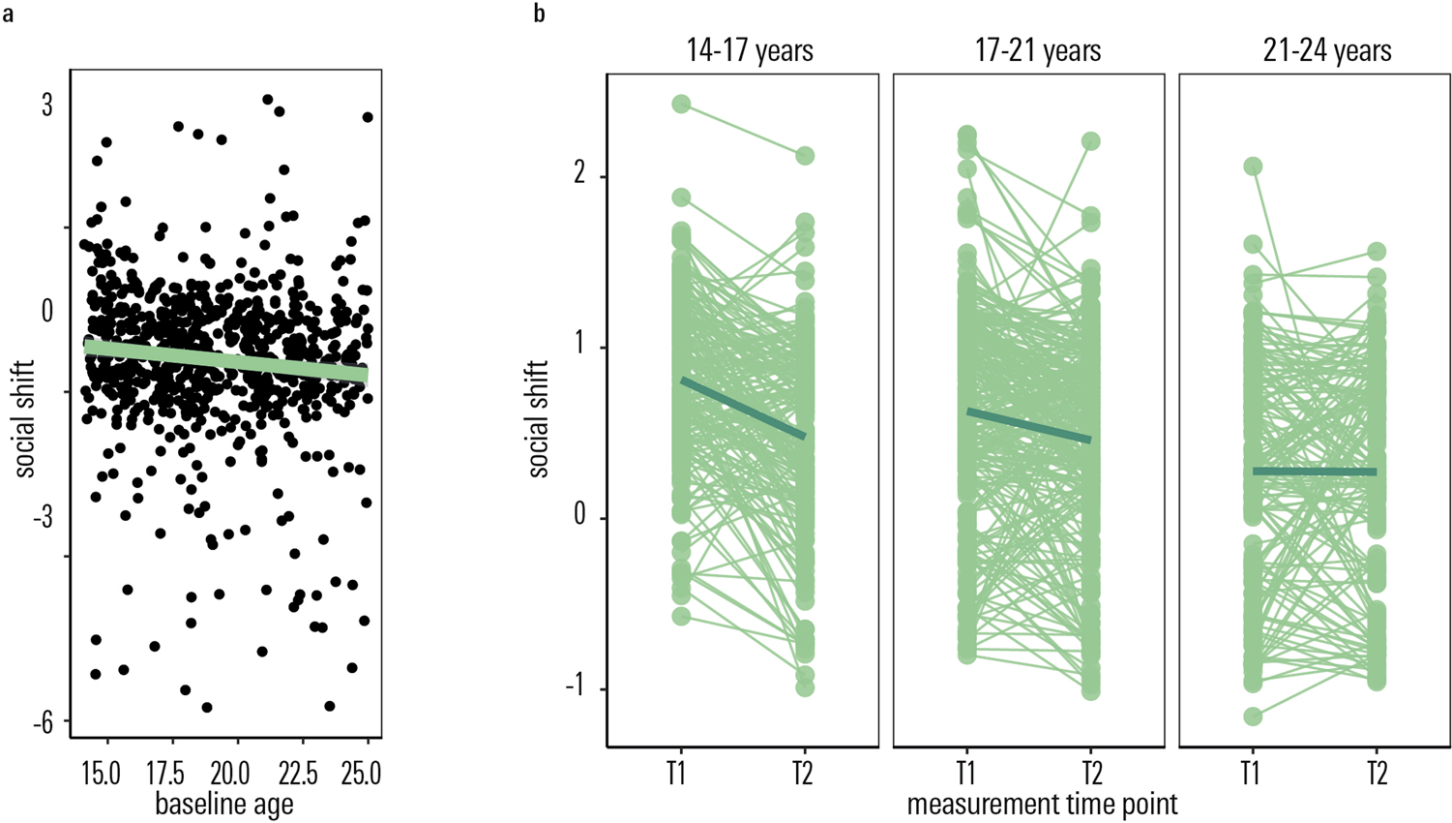
Developmental effects on social susceptibility as measured by preference shifts in the delay discounting task. **a** A tendency to show a peer-induced shift in delay discounting preferences (positive values indicate a change towards the partner) declines with age. The error band denotes the 95% confidence level interval. **b** Such susceptibility to peer influence also decreases within person over the course of the longitudinal follow-up period. We plot posterior estimates from our mixed effects model. Note that baseline age entered the model as a continuous regressor, here we plot 4-year-age bins ≤17 years old, >17 – ≤21 years old, >21 years old, for visualisation purposes alone. *T1* measurement time point 1, baseline assessment; *T2* measurement time point 2, follow-up assessment after ~1–5 years. Source data are provided as a Source Data file.

At baseline, we observed a significant negative association between social susceptibility (indexed as the change in discount rate upon learning about the discount rate of another agent) and age, such that socially induced preference shifts declined with age (*r* = −0.10, *df* = 782, *t* = −2.94, *p* = 0.003, Fig. 2a).

In a longitudinal analysis, we tested whether social susceptibility also changed intra-individually, within the ~1.5 year follow-up period. Indeed, a factor time (baseline vs. follow-up) significantly predicted social susceptibility in our delay discounting task in a linear mixed effects model (F(1,566.64) = 5.11, unstandardised estimate = 0.17, *p* = 0.02), along with baseline age. Social susceptibility decreased intra-individually over the ~1.5 year follow-up period. The interaction of baseline age with time was not nominally significant (F(1,567.06) = 3.78, *p* = 0.05).

In addition, we examined a sample of participants who were also tested 6 months apart (‘short follow-up’). This comprised a sub-sample of *n* = 55 of the total group who came to the lab three times (baseline, 6-month ‘short’ follow-up, 1.5-years ‘long’ follow-up), in the same manner as per our main sample (see ‘Methods’). Repeating the same analysis of longitudinal effects on social susceptibility (i.e. preference shifts in our delay discounting task), in this short follow-up sample, we observed a significant effect of time point (baseline, short follow-up, long follow-up; F(2,104.93) = 6.87, *p* = 0.002). Post-hoc analysis showed that susceptibility decreased significantly over the 18-month period (*t* = 3.47, unstandardised estimate = 0.70, *p* < 0.001), and between 6 and 18 month of follow-up (*t* = 2.88, unstandardised estimate = 0.58, *p* = 0.005), but critically did not do so significantly over the 6-month period from baseline (*t* = .59, unstandardised estimate = 0.12, *p* = 0.56).

### Association of susceptibility to peer influence with the development of peer relationships

In previous work, we observed a positive association of susceptibility to peer influence and sociability in a smaller cross-sectional study, including young adolescents^5^. Here we tested for this association again, including in a longitudinal manner, in our larger dataset. We used a bivariate latent change score model^49^ to test for a co-development of social susceptibility, as indexed by socially induced preference shifts in our delay discounting task, and quality of peer relationships, as measured by the Cambridge Friendship Questionnaire (CFQ^50,51^, freely available at https://osf.io/cf59r/). In a previous study, the CFQ was shown to predict psycho-social resilience in this sample^51^. The model showed that the perceived quality of peer relations increased from baseline to long follow-up (significant intercept of the latent CFQ change score: *z* = 2.28, standardised beta = 0.09, *p* = 0.02, standardised beta). There was no significant covariation of social susceptibility and peer relations at baseline (*z* = −0.77, standardised beta  = 0.03, *p* = 0.44). However, the latent change score model revealed a small, but significant, positive association between social susceptibility, as measured via our delay discounting task at baseline, on rate of change in peer relation development from baseline to the first follow-up. In effect, those who showed a higher tendency to shift their preferences towards their partners’ in our experimental paradigm, also reported larger gains in the quality of peer relations from baseline to follow-up (*z* = 2.12, standardised beta = 0.08, *p* = 0.03). See Supplementary Table 1 for full output of the model.

**Fig. 3:**
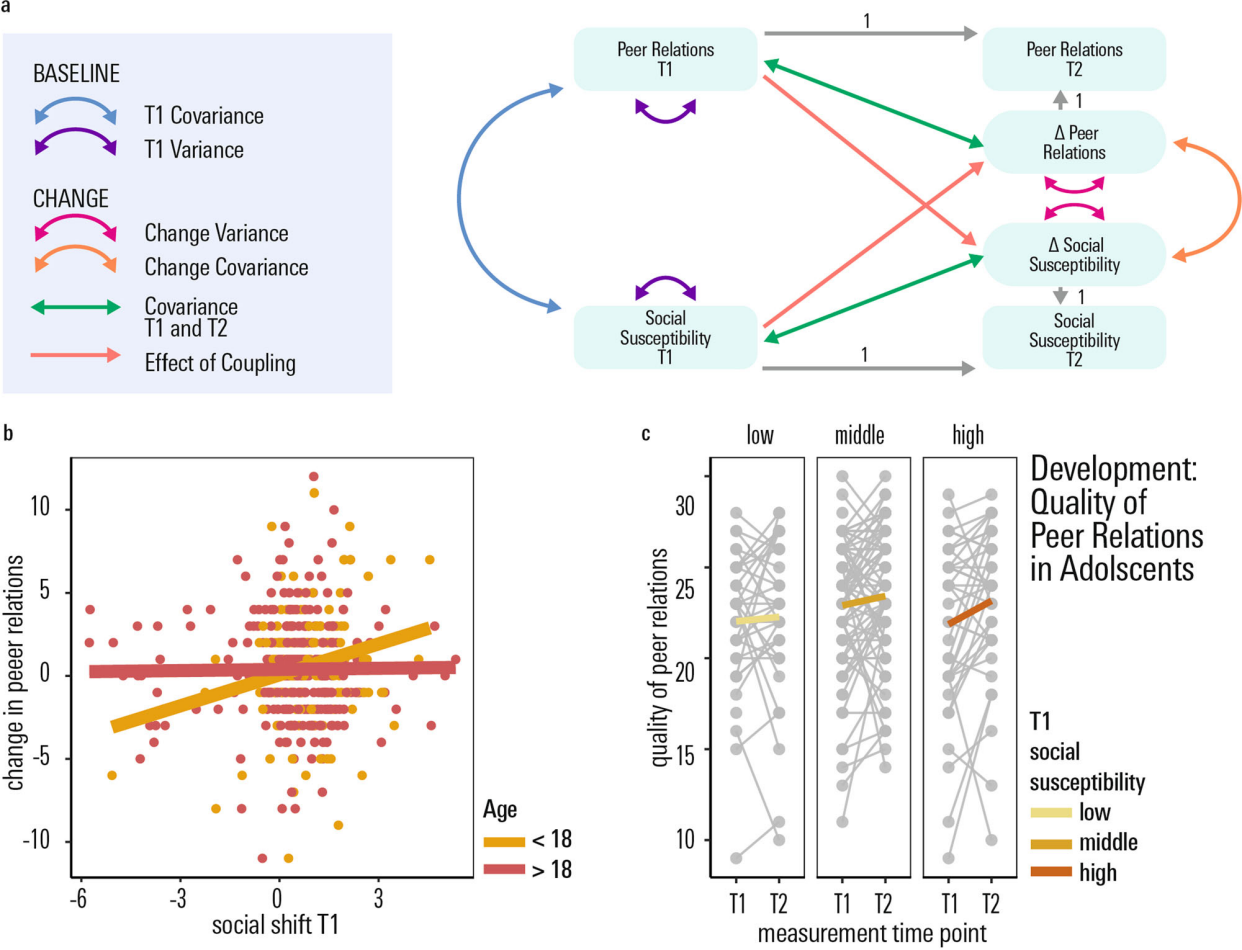
Social susceptibility and the development of peer relations. **a** Schematic of the latent change score model of the longitudinal development of social susceptibility in our task, the longitudinal development of real-life psycho-social functioning (perceived quality of peer relations) from baseline to long follow-up (~1.5 years later) as well as their co-development. Social susceptibility at time point 1 significantly predicts an increase in the quality of peer relationships within the follow-up period. **b** This positive association was driven by the younger (< 18 years old) people in our sample, but was not significant in those aged 18 or older. The full set of parameter estimates is included in the Supplementary Table 1. Error bands denote the 95% confidence level interval. **c** Change in the quality of peer relations in the adolescent subgroup (<18 years), plotted as a function of T1 Social susceptibility. *T1* measurement time point 1, baseline assessment; *T2* measurement time point 2, follow-up assessment after ~1–5 years. Source data are provided as a Source Data file.

Previous findings^4,5^ showed that developmental effects on social susceptibility might be particularly pronounced in younger teenagers. Thus, we repeated the path analysis separately on age specific subsamples of our main sample, namely a younger (participants who were <18 at both baseline and long follow-up, *n* = 153) and an older (participants who were adults, i.e. ≥18 years old, at both measurement time points, *n* = 320) subsample. Comparing this model to a model where the path of interest (social susceptibility at baseline → quality change in peer relations at long follow-up) was constrained to be equal between the younger and older subsample, revealed a significant advantage of fitting age-dependent, sub-group-specific parameters (Log likelihood Ratio Test, Δ*χ*^2^ = 4.47, Δ*df* = 1, *p* = 0.03). This indicates differences in a younger vs. older subgroup regarding the degree to which social susceptibility, as measured with our task at baseline, influences development of peer relations. Analysing the path of interest (baseline task measure of social susceptibility on rate of change in peer relation) separately for the younger (<18 years) and older (≥18 years) subgroup, revealed a significant effect of social susceptibility on social development in the adolescent (<18 years) group alone (*z* = 2.17, standardised beta  = 0.20, *p* = 0.03, see Fig. 3), whereas there was no significant coupling of social susceptibility on social development in the young adult (<18 years) group (*z* = 0.44, standardised beta = 0.02, *p* = 0.68). Results in the younger age group remained significant when controlling for age or sex (all betas > 0.21, all *ps* < 0.04). This suggests that greater susceptibility to social influence earlier in adolescence might be an important factor affecting development of integrative social relationships as we grow up.

A maladaptive trait, which has been extensively discussed in the context of peer influence in the literature^16–19,52^, is substance consumption. Thus, we repeated the structural equation model to analyse an association with the development of substance consumption (alcohol, cigarettes, cannabis). In this generally healthy sample, we did not find any significant association of social susceptibility with substance consumption (all zs ≤ |1.23|, all standardised betas ≤ |0.10|, all *ps* > 0.22).

### Computational modelling: developmental effects on preference uncertainty

One reason for conformity is informational influence, whereby humans use observational information to reduce uncertainty about what to like, even if, as in our task, this does not produce immediate material benefits^31^. We tested the hypothesis that the observed developmental reductions on social susceptibility, indexed by preference shifts in delay discounting towards the direction of the experimental partner, occur as a consequence of developmentally decreasing uncertainty about own preferences (‘preference uncertainty’). Thus, in a next step, we used a previously validated formal computational model^34^ to estimate an individual ‘preference uncertainty’ parameter (See Supplementary Information for effects of other parameters of the model). This allowed us to test for developmental effects on preference uncertainty and whether this accounts for the observed susceptibility to social influence.

Preference uncertainty was significantly associated with social shift at both measurement time points, consistent with an informational account of conformity (all *rs* > 0.50, all *ts* > 15.57, all *ps* < 0.001, see Supplementary Fig. 5).

**Fig. 4:**
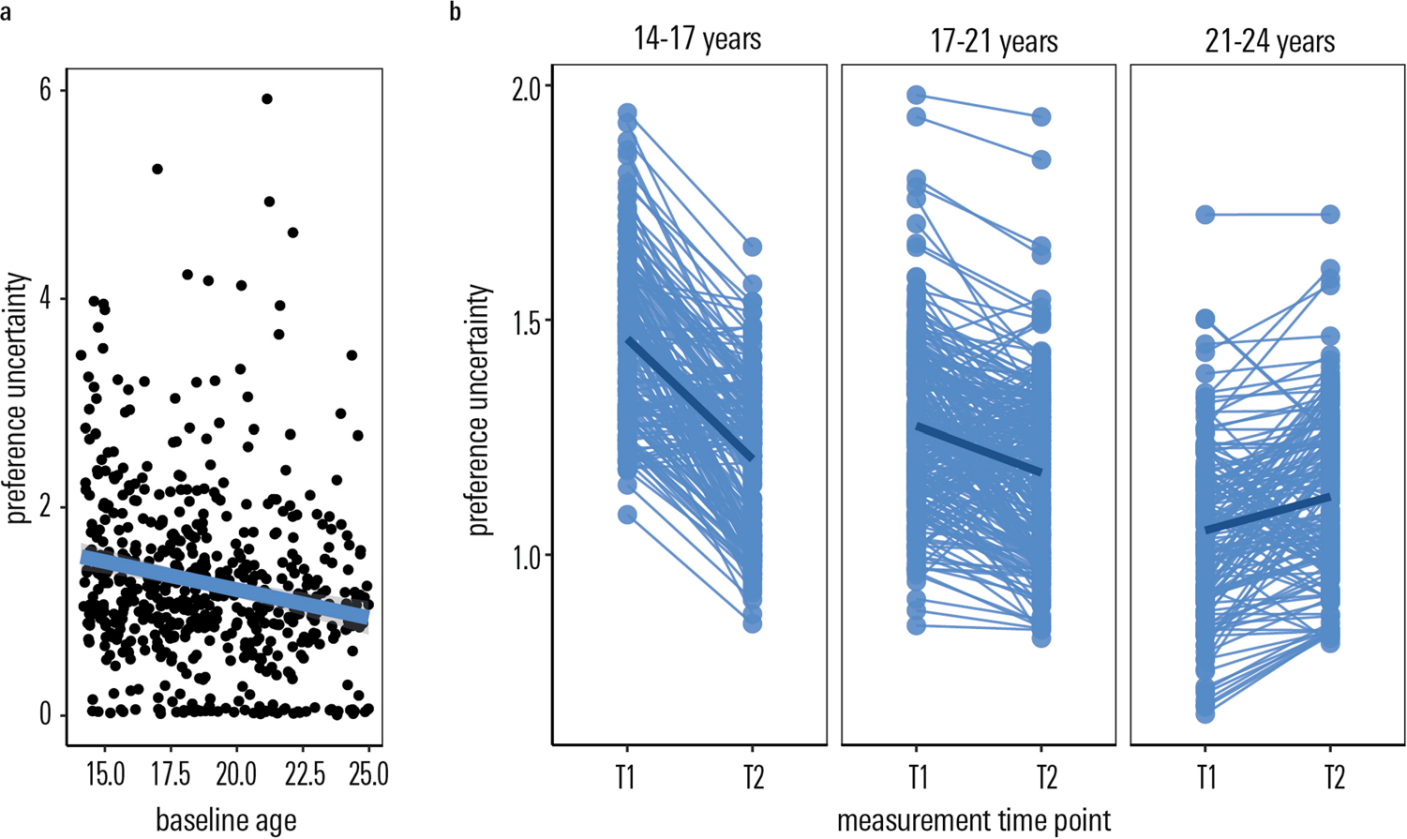
Development of preference uncertainty. Preference uncertainty decreases cross-sectionally (**a**) and over the 1.5-year follow-up (**b**). This decrease is most pronounced in the youngest participants (in terms of baseline age). We plot posterior estimates of our mixed model analysis. The error band denotes the 95% confidence level interval. Note that baseline age entered the model as a continuous regressor, here we plot 4-year-age bins ≤17 years old, >17 ≤21 years old, >21 years old, only for visualisation purposes. *T1* measurement time point 1, baseline assessment; *T2* measurement time point 2, follow-up assessment after ~1–5 years. Source data are provided as a Source Data file.

At baseline, preference uncertainty negatively correlated with age (*r* = −0.15, *t* = −4.36, *df* = 780, *p* < 0.001, Fig. 4a).

A longitudinal linear mixed effects model revealed a significant effect of baseline age (F(1,564.53) = 14.22, *p* < 0.001), time (F(1,564.86) = 5.69, unstandardised estimate = 0.11, *p* = 0.02), and an interaction of baseline age and time (F(1,565.41) = 9.29, unstandardised estimate = 0.05, *p* = 0.002) on preference uncertainty. To visualise the latter interaction, we plot longitudinal (intra-individual) change as a function of age at baseline in Fig. 4b. Figure 4b suggests that relevant longitudinal changes in preference uncertainty were strongest in those that were ≤17 years of age at baseline.

In addition, we again analysed the reduced sample of participants who were also tested 6 months apart (‘short follow-up’). In line with findings in our main study sample, we observed a significant effect of time on preference uncertainty (F(2,105.29) = 3.57, *p* = 0.03) in the ‘short follow-up’ subsample. Post-hoc inspection revealed that preference uncertainty decreased within-person over the three measurement time points in the reduced subsample. Post-hoc tests showed that a contrast of first and last (1.5 year follow-up) time points was significant (*t* = 2.67, unstandardised estimate = 0.33, *p* = 0.009), whereas neither the change between baseline measurement and 6-month follow-up was significant (*t* = 1.23, unstandardised estimate = 0.149, *p* = 0.22), nor the change between 6-month and 1.5 year follow-up (*t* = 1.44, unstandardised estimate = 0.18, *p* = 0.15). This pattern of findings—more pronounced effects after a greater period of time had elapsed—seems inconsistent with changes being due to a mere training effect, as in this case we would expect a stronger change after 6 compared to 18 months.

### Mediation analyses: preference uncertainty accounts for developmental effects of social susceptibility

**Fig. 5:**
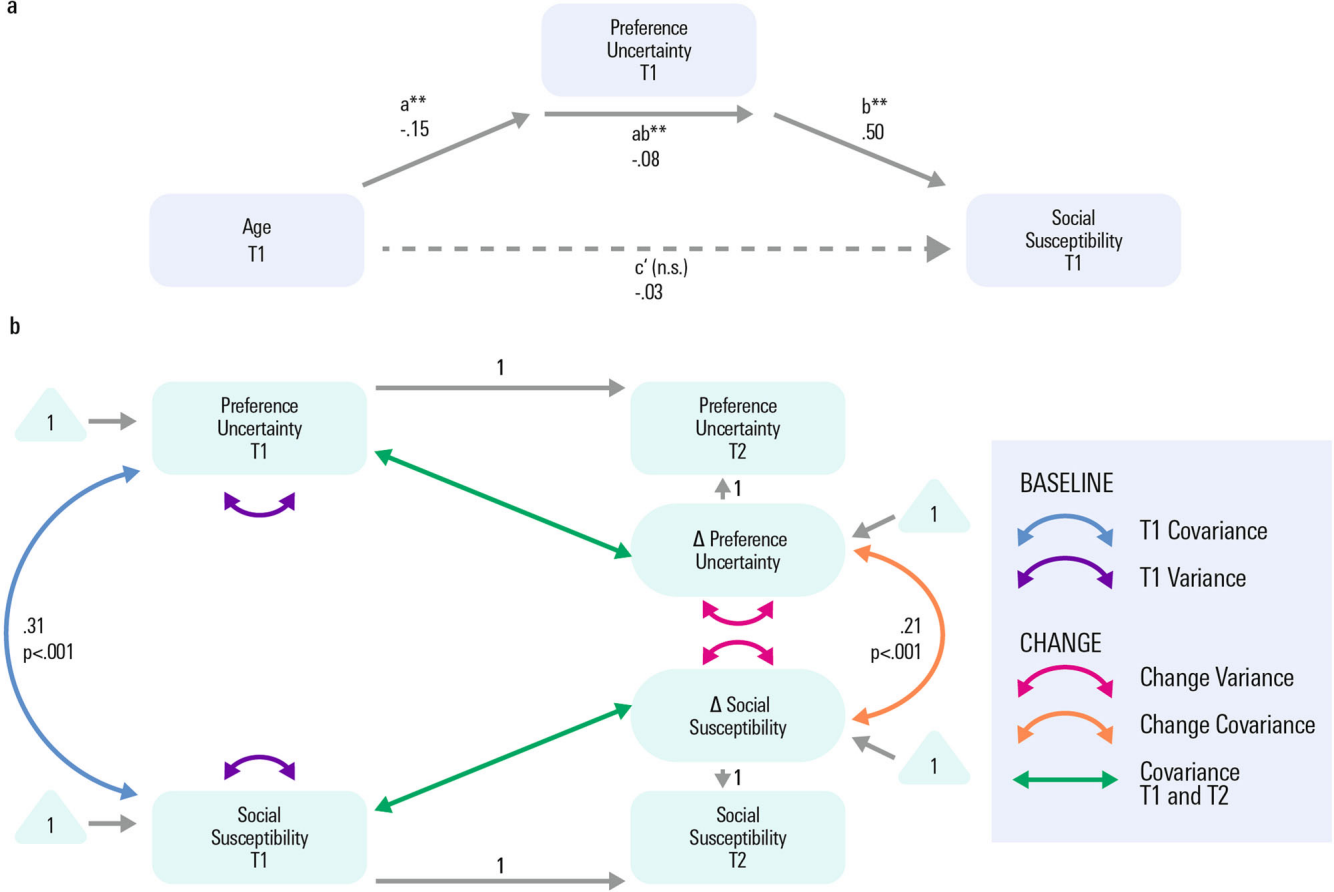
Mediation analyses testing for a mediating role of preference uncertainty for the relationship of development with social susceptibility. **a** Mediation analysis for social susceptibility (i.e. preference shift measured in our delay discounting task) as predicted from age and mediated by preference uncertainty at T1. Age predicted preference uncertainty (path a). The mediator (preference uncertainty) predicted preference shift (path b, controlled for the age effect on preference uncertainty). Importantly, the mediation effect was significant (path ab). The direct path c’, namely the age effect on preference shift after accounting for the mediation, was not significant. The proportion of total variance accounted for by the mediation effect was significant. Thus, the age effect on social susceptibility at baseline was accounted for by preference uncertainty. **b** In line with our assumption that development of social susceptibility is accounted for by development of preference uncertainty, the bivariate latent change score model not only showed a significant covariation of preference uncertainty with social susceptibility at T1, but also significant covariation of the rate of longitudinal change in both domains. The full set of parameter estimates is included in Supplementary Information Table 2. *n.s.* not significant, T1 measurement time point 1, baseline assessment; *T2* measurement time point 2, follow-up assessment after ~1–5 years.; ** denotes *p* < 0.001 Source data are provided as a Source Data file.

Our computational model of social shifts posits a specific mechanism by which social influence arises, namely a reduction in one’s own preference uncertainty by learning how someone else performs a task^31,34^. As preference uncertainty declines with age, and decreases longitudinally within person, this raises a possibility that age-related changes in social susceptibility (as found here in the paradigmatic case of a delay discounting task, as well as in previous studies using different behavioural paradigms) is driven by the age-related change in preference uncertainty. To test this, we first set up a model where we tested a possible mediation of the cross-sectional age effects on preference shift by preference uncertainty on baseline data. We found that an effect of age on preference shift in delay discounting was accounted for by the mediating effect of preference uncertainty, corresponding to a significant full mediation (significant proportion of mediation^53^: *z* = 3.03, standardised beta= 0.723, *p* = 0.002, Fig. 5A). That is, the significant age effects on social susceptibility, as found here and in many previous studies, are, in this study, explained by age effects on preference uncertainty.

It is important to note valid concerns regarding cross-sectional mediation analyses and the degree of causal inference that can be drawn from them^54^. Here, we cautiously apply these analyses building on the temporal order of our task, as well as theoretical assumptions on informational influence including recent experimental studies demonstrating a role for uncertainty as a driving influence^55^.

In a next step, we examined the covariation of longitudinal change in preference uncertainty with longitudinal change in social susceptibility, using latent change score modelling. To do so, we changed the autoregressive and coupling effects to co-variances, to display and model the unconditional change scores. We observed a significant covariation of rates of change in both parameters (*z* = 3.82, standardised beta = 0.205, *p* < 0.001, Supplementary Table 2), in line with our assumption that development of social susceptibility is accounted for by development of preference uncertainty.

### Co-development of brain structural correlates and preference uncertainty

To establish whether there is a structural neural correlate for developmental effects on preference uncertainty, we used bivariate latent change score modelling testing for the co-development of cognitive and brain structural development in a subsample of subjects who underwent both our experimental task sessions and structural MRI (at baseline and after ~1.5 years, *n* = 186, see ‘Methods’). In a hypothesis-driven approach, we focussed on myelination, given its pivotal role in adolescent brain development and plasticity, and building on recent findings on its association with trait measures in adolescent development^39^. Medial prefrontal cortex (mPFC) myelin development has been demonstrated to be associated with socially relevant behaviour in several previous rodent^42–45^ and human^41,46^ studies. In a similar discounting task, utilising repetition-suppression to assess neural plasticity during fMRI, we previously showed that mPFC is the principal region expressing within-task plasticity as preferences shift^36^. We, therefore, focused on mPFC as a key region of interest, testing whether a myelin marker in this region relates to developmental effects on preference uncertainty. See Supplementary Information methods for pre-processing of the MRI data.

**Fig. 6:**
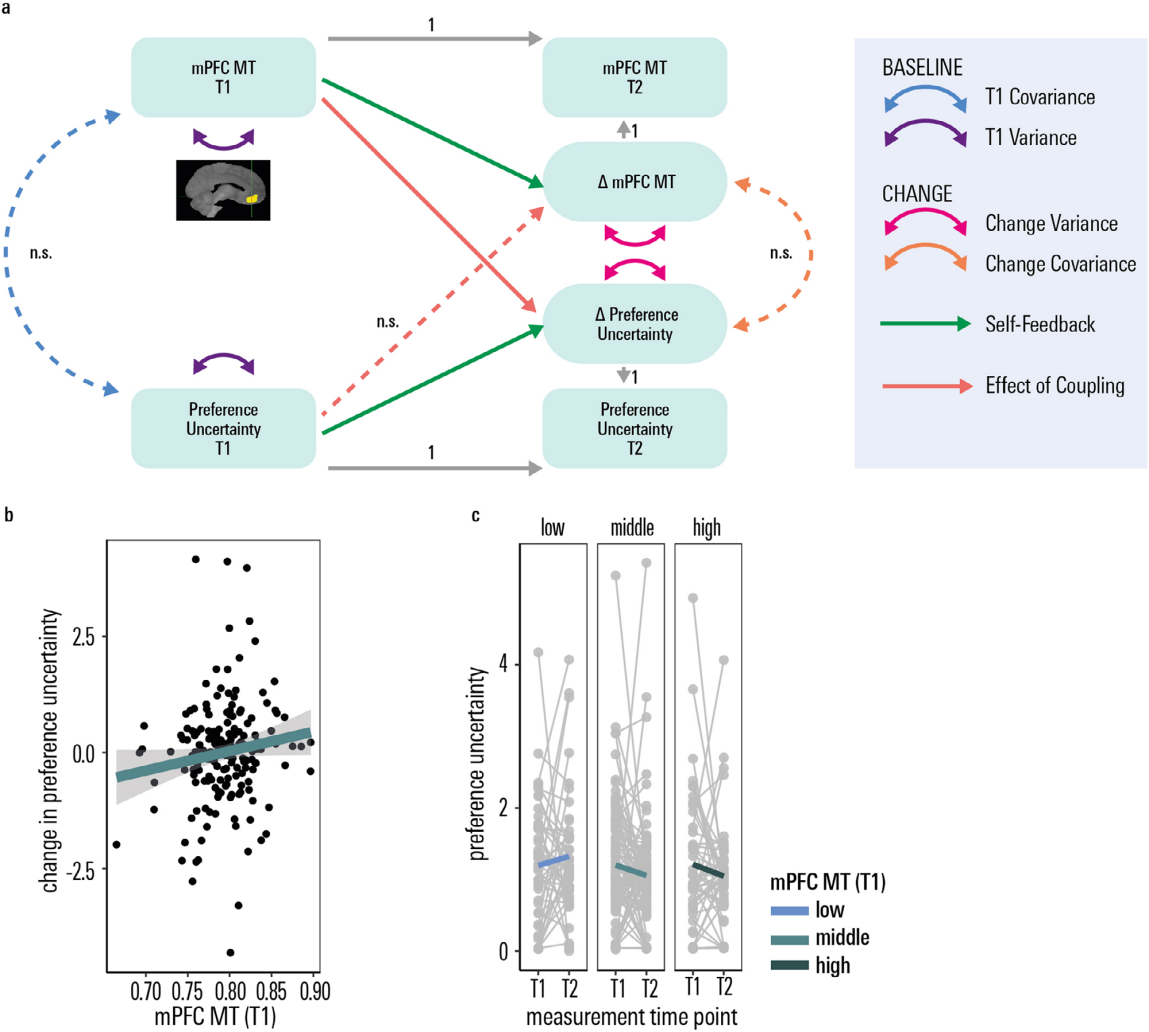
Association of preference uncertainty with the mPFC myelin marker. **a** Bivariate latent change score model. Three developmental brain-behaviour relationships are possible and tested for by the model: (1) differences in myelin at baseline affect the rate of preference uncertainty decrease; (2) preference uncertainty at baseline predicts the degree of myelin gain between baseline and long follow-up, (3) correlated change (the degree of reduction in preference uncertainty is correlated with the degree of myelin marker change). While the path indicating the mPFC myelin marker (magnetisation Transfer) as a significant predictor of longitudinal change in preference uncertainty was significant (standardised beta = −0.13, *p* = 0.03), the other cross-domain coupling paths were not. Solid lines: significant path, dashed line: non-significant path. No means are displayed for clarity; the full set of parameter estimates is included in Supplementary Table 3. **b** Higher values of the mPFC myelin marker (magnetisation transfer) at measurement time point 1 led to a more pronounced longitudinal change in preference uncertainty (note that a stronger longitudinal decline is coded as positive (‘more change’) for illustration purposes). The error band denotes the 95% confidence level interval. **c** Longitudinal decrease in preference uncertainty as a function of different levels of T1 myelin marker values. For illustration purposes, we plot preference uncertainty residuals corrected for baseline age, IQ and sex. *n.s.* not significant, *T1* measurement time point 1, baseline assessment; *T2* measurement time point 2, follow-up assessment after ~1–5 years, *mPFC* medial prefrontal cortex, *MT* Magnetisation Transfer. Source data are provided as a Source Data file.

Using an anatomically defined mPFC mask (based on the Harvard-Oxford atlas, see Supplementary Information), we extracted estimates of a myelin-sensitive marker, Magnetisation Transfer saturation (MT). This allowed us to investigate a cross-domain coupling that captures the extent to which change in one domain is a function of the starting level in the other using latent change score modelling (model fit indices: p_Chi2_ = 0.74, CFI = 1, RMSEA = 0.00, SRMR = 0.02). Sex, IQ and scanning site were added to the imaging analysis a priori as they were previously found to be associated with myelin-related measures^39,40,56,57^. We observed that baseline intra-cortical mPFC MT was predictive of the longitudinal change in preference uncertainty (*z* = −2.14, standardised beta = −0.13, *p* = 0.03, Fig. 6), i.e. more intra-cortical mPFC myelin (as approximated via MT) lead to a more pronounced reduction in preference uncertainty over the ~1.5 year follow-up.

Preference uncertainty did not significantly predict longitudinal brain development, and there was no covariance in rates of change in both domains (all standardised betas ≤ 0.10 all *ps* ≥ 0.13, see Supplementary Table 3 for full output of the latent change score model). We performed a control analysis using an ROI chosen on account of its high developmental relevance, based on our previous findings that in this cohort age-related and longitudinal changes in MT show a peak in angular gyrus^39^. Repeating the same latent change score model in a control region (based on a Harvard-Oxford atlas defined ROI of angular gyrus, model fit indices: p_Chi2_ = 0.83, CFI = 1, RMSEA = 0.00, SRMR = 0.02), we did not observe any significant brain-behaviour coupling (all standardised betas ≤0.10, all *ps* ≥ 0.12).

## Discussion

In adolescence, a greater susceptibility to social influence is considered a driver of maladaptive real-life behaviour (e.g. drinking, reckless driving, delinquency, self-harming behaviours)^12–15,18,19^. Here, using a longitudinal design involving a large cohort of 14–24 year-olds, combined with a social delay discounting paradigm as well as quantitative brain imaging, we replicate a finding of increased social susceptibility in adolescents and characterise its computational and neuro-developmental correlates.

We replicate previous developmental effects on social susceptibility in the paradigmatic case of a delay discounting task, showing that social susceptibility decreases with age from adolescence to adulthood. We extend on this previous finding in a number of ways. First, we provide longitudinal evidence for a developmental decrease in susceptibility to social influences. Second, we show that our task-based measure of social susceptibility in young healthy adolescents predicts longitudinal improvements in peer relationships. Third, we outline cognitive and computational correlates for these effects, providing evidence in line with the idea that higher social susceptibility in younger adolescents may be explained by this population being more uncertain about their delay discounting preferences, thereby rendering them more prone to adopt others’ preferences. Over the course of development this ‘preference uncertainty’ decreases, which may in turn attenuate a need, and the potential impact of, social influences on one’s own behaviour. Last, we demonstrate a candidate neuro-developmental correlate of this effect by showing that a myelin-sensitive marker within mPFC, a key region mediating social preference shifts, predicts longitudinal change in preference uncertainty.

We replicate our previous finding, from a young adult sample, showing delay discounting preferences can be systematically changed by learning about another’s delay discounting preferences^36,47^. Extending these findings, we now show that the degree of a preference shift is, in this sample of 14–24 year-olds, most pronounced during younger adolescence, a developmental period characterised by significant social-affective transformations^1^. Importantly, our longitudinal design allowed us to demonstrate a within-person developmental decrease in this social susceptibility. A separate analysis in a retest sample showed that these observations are not likely to be due to a training effect.

Importantly, we found longitudinal evidence that higher social susceptibility at a younger age might have an important adaptive, rather than maladaptive, role in this healthy population. This adds to emerging evidence which implicates the role of peers in adolescents’ positive adjustment as well as in prevention programmes that aim to foster adaptive development^23,50,58,59^. Exploiting our accelerated longitudinal design^39,49,51,60^, we demonstrate that the developmental improvements in real-life psycho-social functioning, assessed by the perceived quality of peer relations, is predicted by higher susceptibility to social influence, in terms of a higher tendency to shift towards another person’s delay discounting preference, particularly in younger teenagers. This extends on cross-sectional findings in healthy younger adolescents, which showed that behavioural contagion can be associated with a higher degree of social integration^5^. Notably, in previous accounts, susceptibility to social influence was mostly highlighted in the context of maladaptive real-life behaviours in teenagers. For example, both real-life and digital peer influence is suggested to lead to higher rates of delinquency, real-life risk-taking, unprotected sexual intercourse, substance consumption and self-harming behaviours^12–19,21,22^. In this study of healthy adolescents, however, our experimental measure of susceptibility to social influences did not relate significantly to substance consumption, nor did it predict such maladaptive behavioural tendencies in a longitudinal fashion. This discrepancy might suggest that the impact of higher susceptibility to peer influence in teenagers depends on the very nature of social influence, including the nature of the role models who exert this influence. In our sample of relatively healthy teenagers, being behaviourally responsive to peers indeed led to successful social adaptation in real life, a key requirement of adolescent development. However, even though in our sample quality of peer relations (measured by the CFQ) has been shown to predict psycho-social resilience in a previous study^51^, a higher degree of social integration might, in maladaptive environments, lead to harmful behaviours (e.g. closer social relationships accompanied by increased ‘social’ smoking and drinking). Our study did not recruit young people from the most deprived backgrounds, or having significant psychopathology, hence our results cannot be safely extrapolated to these groups, who may be subject to higher exposure to maladaptive influences. Further research could assess whether the extent to which susceptibility is adaptive depends on the type of environment an adolescent grows up in.

Finally, using computational modelling, we provide a mechanistic account for the frequent previous observation of adolescents being more prone to peer influence than adults (e.g.^3–5^). In principle, two routes to conformity are possible, normative influences (adhering to social norms and expectations to gain interpersonal benefits such as being part of a group), and informational influences (reducing uncertainties about the world and the self by observing others)^31^. In the existing literature, peer influences on decision-making are generally implicitly interpreted within a normative account of conformity. Whilst we do not question the importance of normative influences during adolescence, here we specifically probed the latter hypothesis, using a Bayesian model, previously validated on this same task^34^. We show that uncertainty about one’s own preferences (here, in the paradigmatic case of delay discounting) predicted social shift, and that developmental differences in susceptibility to social influences are mediated by decreasing uncertainty as participants aged. This provides a novel interpretation of our current and previous findings. Intriguingly, this interpretation again places susceptibility to social influence into an adaptive context—as a rational means to reduce one’s own uncertainty. Given that we cannot directly derive causality from our findings, an alternative interpretation for the longitudinal co-development of both a reduction in preference uncertainty and a decrease in social susceptibility is, however, the possibility that decreasing susceptibility to social influence causes individuals to be more certain in their preference. However, a role for uncertainty driving social influence effects has recently been shown in a study experimentally manipulating uncertainty in young adults^55^. To gain greater insight into the directionality of developmental effects, future studies of adolescent development might usefully manipulate both informational and normative sources of conformity to disentangle their respective effects on adolescent behaviour, as well as test for potential interactions between these processes. For example, is social influence on adolescents highest when they are uncertain and feel an enhanced need to conform in order to be accepted by peers? The computational model applied here might prove useful for this question, as it enables modelling of relevance of the source of social influence in addition to preference uncertainty.

In the context of our computational modelling analysis, a limitation is that model fit correlated with age, such that younger participants were fit less well by our computational model. In some sense, this is inherent in our research question, as the key variable we examine, preference uncertainty, drives choice variability. Our finding of both decreasing preference uncertainty and increasing model fit with age reflect that behaviour is less variable in older subjects within our sample. Varying goodness-of-fit of a specific model is a challenge for developmental modelling studies^61,62^ and may be indicative of either varying stochasticity or different cognitive processes underlying task behaviour at different ages. Potential solutions are now emerging in the literature, including novel data analysis strategies (e.g. data-driven approaches^63^) or experimental design specifically tailored to disentangle random from directed choices across development^64^.

In addition to preference uncertainty, our study also showed that features of the observed other influence how much young people shift towards this other’s preference. Thus, social susceptibility was stronger towards the other person if they were more patient than oneself. This fits with findings in the domain of risk preferences, which highlight that in an experimental set-up where choices were not observed, those in late adolescence are more influenced by safe than by risky information^6,24^.

Further limitations of this study relate to ecological validity, in that a social influence was implemented by a computerised agent rather than a real social interaction partner. The study did not include a non-social control condition. However, the experimental design followed closely our previous study in young adults of an age range overlapping with the older half of our sample^36^. That study included additional control experiments that provided evidence that social influence effects in this task cannot be explained by simple stimulus- or action-based reinforcement, but are depended on mentalizing^36^. On this basis we consider our conclusions are likely to remain valid, even if participants are influenced by anthropomorphic digital agents. Indeed, influence by digital agents seems particularly relevant in the context of adolescent development, where evidence points to this age group being susceptible to influence from digitalised social input via (social) media (including bots, computer algorithms and fictive characters in video games and series). These influences have recently been shown to exert a deleterious impact on adolescents’ and young adults’ mental health^22,65,66^ notwithstanding potential positive influences and impact on prevention^67^.

In our structural brain imaging analysis, we uncovered a neural correlate of preference uncertainty. MPFC myelin development has repeatedly been shown to be associated with social cognition and socially relevant behaviour^42–45,41,46^. Using the same social delay discounting task, during functional imaging, we have shown that mPFC expresses neuronal plasticity that predicts preference malleability^36^. Myelin maturation unfolds throughout adolescence and into young adulthood and is a key mechanism underlying neuronal plasticity^37,68^. This motivated a hypothesis that a marker of myelin in the mPFC would relate to developmental effects on preference uncertainty. Whilst we were primarily interested in the co-development of preference uncertainty and mPFC maturation, especially the unfolding of myelination based on an a priori hypothesis, we acknowledge that mPFC myelination is likely to be associated with other cognitive or socio-emotional functions, as for example shown previously in rodents^69,70^. Indeed, we found that baseline MT in this region was predictive of a greater reduction in preference uncertainty over time. In contrast, baseline preference uncertainty did not predict changes in MT, and there was no association between the rates of change in both measures. This suggests that the observed longitudinal reduction in preference uncertainty over time is accelerated when myelin in the mPFC is at a more mature absolute level. This finding suggests that structural differences in mPFC myelin precede cognitive development of preference uncertainty, a brain-behaviour dynamic that is also referred to as ‘structural scaffolding’ in developmental cognitive neuroscience^71^. It is compatible with an hypothesis that the current state of the brain (here, MT-related myelination) provides, in some sense, preconditions that facilitate cognitive growth^71^. The absence of a cross-sectional brain-behaviour correlation highlights that the level of mPFC myelin is not reflective of absolute behavioural maturity, but rather reflects a potential for subsequent growth, consistent with myelin’s role in plasticity. Furthermore, this effect is independent of baseline age, pointing towards inter-individual differences in the timing of this maturational process. These findings underscore the importance of brain structural maturation in cognitive development during adolescence.

Notably, the effect size of the developmental effects on susceptibility to social influence, as well as their association with peer relations or neuro-developmental markers, was lower than might be expected from theoretical accounts and lower sample size studies. This is consistent with reports on large-scale replication efforts of original findings in psychological science in bigger sample sizes, which reported only effect sizes on average of 1/2 the originally reported effects^72^. On the one hand, it highlights the need for larger sample sizes and replication in order to estimate meaningful effect sizes in the field of developmental psychology. As this is not a direct replication study of a previously reported investigation, it may, however, also be dependent on precise methodological details and the specific demographics of our sample, e.g. on the fact that our sample did not include very young adolescents for whom strong susceptibility effects have been reported previously^4,5^ (but also note^73^). Our findings stress the importance of longitudinal designs for developmental psychology. Indeed, the association of peer susceptibility with the quality of real-life peer relations, as well as neuro-developmental markers, were, albeit with moderate effect sizes, only observed within-person, but not across-participants, potentially due to the higher power of within-subject designs as compared to between-subject designs.

In sum, our study showcases the role of computational modelling and large-scale, longitudinal developmentally sensitive studies^6,74^, identifying the psychological mechanisms and neuro-developmental processes which underpin the phenomenon of susceptibility to social influences over adolescent to young adult development.

## Methods

### Main sample

The experimental task was delivered as part of a task battery administered to a sample of community dwellers between the ages of 14 and 24 in Cambridgeshire and London, as part of the Neuroscience in Psychiatry Network (NSPN) project^60^. Participants 16 years of age and above provided written informed consent. Participants below this age provided assent, and their parent (or legal guardian) provided written informed consent. The Cambridge Central Research Ethics Committee approved the study (12/EE/0250). Data for this task was available from *n* = 784 (401 female) participants for baseline. *N* = 738 of this baseline data has informed a previously published computational model validation paper^34^. Participants were 14.10–24.99 years old (mean = 19.05, *sd* = 2.96) at baseline. *N* = 569 (284 female) participants returned for a second assessment ~1.5 years later. Mean age at follow-up was 20.29 years (range: 15.11–26.48 years, *sd* = 2.97) while mean time between first and second assessment was 1.48 years (range: 0.98–2.62 years, *sd* = 0.30). Structural imaging and task data were available (and passed quality assessment) for *n* = 186 participants for both measurement time points (91 females).

### Retest ‘short follow-up’ subsample

A subsample of *n* = 55 participants completed the task three times, with an additional interim session after a ~6-month follow-up period. Considering all three measurement time points in this subsample allows us to approximate whether observed longitudinal differences are merely due to retest effects (i.e., practice effects, familiarity with the task). Even though changes after 6 months could also reflect development (during rapid periods of adolescent maturation)^75^, here we hypothesised a pattern of results consistent with a developmental (rather than a retest) account, that would show a stronger effect over the long-term (after ~1.5 years) than over the short-term (after ~6 months).

### Task

We used a social version of a delay discounting task (Fig. 1,^34,36^). The task was programmed in MATLAB 2012a using the Cogent graphic toolbox (http://www.vislab.ucl.ac.uk/Cogent/). The task consisted of three phases. In phase 1, participants played 60 trials of a temporal discounting task where they had to decide whether to choose between a smaller amount of money paid out immediately or a larger amount paid out at an indicated time in the future. Phase 1 decisions were used to determine their initial value k_phase1_ in a standard hyperbolic discounting model^76^:

1 $${V}_{D}=\frac{{R}_{D}}{1+{kD}}$$

where ${V}_{D}$ is the delay-discounted value of a reward, *R* is the reward, *D* is the delay, and *k* is the hyperbolic discounting parameter. Based on empirical observations that the population follows approximately normal distributions in log *(k)* space, all reported analyses are based on log (*k*)^34,36,77^.

The 60 trials of phase 1 included 30 offer pairs from a standard set covering a wide range of values of k. Half of the trials were an interleaved set of 30 from an adaptive algorithm which calculated a probability distribution over possible values of k and then selected a pair of options likely to reduce the entropy of that distribution as much as possible (see^34^ for details). Participants were instructed to respond according to their own true preferences.

In phase 2, a second player was introduced. Participants were instructed to make choices in the same delay discounting task for the other player so as to learn the discounting preferences of the other. This preference of the other person was based on the baseline preference of our participant. In a between-subjects manner, the observee’s delay discounting preferences was manipulated such that the other was chosen to be either more or less patient than the participant himself. More specifically, in 2/3 of the cases, the observee was chosen to have k_other_ shifted from k_self_phase1_ by one standard deviation towards the mean of the population distribution, and in 1/3 of cases away from it (in log space). This procedure was repeated for the longitudinal administration of the task, such that the direction of the preference of the other (more vs. less patient than our participant) could be either the same or different for baseline vs. follow-up. Participants received feedback as to whether their choice on behalf of the other was correct in terms of the other’s discounting preference. where correct choices were defined using a simulation of the other’s choice based on their discounting preference. In case the participant’s response matched the simulation’s prediction, the choice was coded as correct. These ‘learning about the other’ trials were presented until either the participant got eight correct answers out of the most recent 10 (minimum: 20 trials), or until 60 learning trials were completed. See Supplementary Results for additional analyses of learning performance.

In phase 3, we interleaved mini-blocks of 10 trials ‘choose for self’, which were as in phase 1, and 10 trials ‘choose for other’, in order to keep the other’s discounting preference. This allowed us to estimate social shift scores *(log k*_self_phase3_*– log* k_self_phase1_) that evaluate a change in delay discounting preference pre- vs. post learning about the other, and thus inter-individual differences in susceptibility to social influence. Note that age remained significantly associated with social susceptibility, also when using a relative measure of preference shift (log *k*_self_phase3_ – log k_self_phase1_) / log k_self_phase1._

We informed participants that one of the ‘choose for self’ trials from the entire task would be chosen at random and the choice they made paid out for real at the appropriate delay. Participants were instructed that there was no financial incentive to make correct choices in the ‘choose for other’ trials. Note that this study was based on a previous study in our lab^36^ which included two carefully designed control conditions, providing evidence that participants in this task mentally simulate the other agent. In this previous study, the first control condition tested an alternative hypothesis that behavioural shifts simply reflect stimulus/action-based reinforcement or priming effects. This control condition consisted of the same stimuli and actions, but a framing of another agent with inter-temporal preferences was removed (see ‘Methods’ and Supplementary Results in^36^ for details). There, we demonstrated that no behavioural shifts occur in response to the other in this control condition, despite exposure to mathematically equivalent choices and similar outcomes. On the contrary, when participants played with an anthropomorphic computer agent that showed inter-temporal preferences, we found no significant difference in preference shift rates compared to a human partner. This constitutes evidence that, in our context, the ‘active ingredient’ of social influence most likely lies in ‘mentalising’ or ‘Theory of Mind’^78,79^ in relation to the other agent, rather than their physical nature.

### Computational modelling

Our model was first introduced and validated on the majority of baseline datasets of this study in^34^.

#### Rationale

The focus of our study here was to investigate the neuro-developmental evolution of social susceptibility. Here we could exploit the analytical tool derived from our previous work, well suited to test neuro-developmental hypotheses regarding delay discounting, preference uncertainty relevance of the other^34^. In short, we adopt a Bayesian approach to model a change in belief in one’s own delay discounting preferences as a function of (i) ‘preference uncertainty’ (as reflected in a participants’ choice variability, in simple terms this captures how uncertain a person is, in the delay discounting task, about their own preferences *prior* to any social exposure in the task); and (ii) the relevance of the social influence source.

We describe participants as holding uncertain beliefs $p\left(k\right)$, modelled by a Gaussian distribution, over their log-discounting coefficient. Thus, they are uncertain about their discounting preferences, sampling a new value from their belief distribution at each trial. The width of this belief distribution captures a subject-specific degree of preference uncertainty. The model further assumes that both the subject and the social partner come from the same reference distribution $N\left(k,{\sigma }^{2}\right)$, the width of which describes the ‘relevance of the other’ and is a fitted parameter of the model. By observing the preference choice data of the other, *d*_*O*,_ subjects can update their own preference belief distribution $p\left(k\right)$ in light of what they learn about the other. In this Bayesian formulation, the more uncertain subjects are about their preferences (the more choice variability they show in the absence of social influence), the more they shift after learning about others. Thus, this model formalises the notion of informational conformity, namely conforming with others to reduce one’s own uncertainty. Please refer to our previous model validation study^34^ for the algorithmic implementation. There we found, based on model selection, that decision variability during the task was best described as reflecting uncertainty about discounting preference, as opposed to decision noise added after evaluation (as, for example, in the softmax rule). See Supplementary Information for parameter recovery results.

### Structural MRI

Of the participants who had completed the task twice, *n* = 186 (after quality assessment) were also scanned twice (baseline and 1.5 years follow-up) on identical Siemens Magnetom TIM Trio whole-body 3 T MRI scanners in Cambridge and London as per the quantitative multi-parameter mapping (MPM) protocol. This included a whole-brain multi-echo FLASH magnetisation transfer weighted contrast at 1 mm isotropic resolution (TR: 23.7, *α* = 6°, 176 sagittal slices, FOV = 256 mm × 240 mm, matrix = 256 × 240 × 176). Quantitative magnetisation transfer saturation (MT) maps were derived using biophysical models with the hMRI toolbox (www.hmri.info) for SPM (Wellcome Centre for Human Neuroimaging, London, UK, http://www.fil.ion.ucl.ac.uk/spm). These maps have been shown to correlate highly with histological measures of myelin^80,81^. Based on our a priori hypothesis, we created an anatomically defined mask of the mPFC based on the probabilistic Harvard-Oxford cortical structural atlas (thresholded at 30%). Mean MT values from within this mask region were extracted from each map using FSL (www.fmrib.ox.ac.uk/fsl/) as a proxy for intra-cortical mPFC myelination. See Supplementary Information for more details with regards to MRI pre-processing, quality assessment, and control analyses.

### Statistical analysis

All data were analysed using R 3.4.3^82^ with R Studio Version 1.1.383^83^. Mixed models for longitudinal analyses included a categorical within-subject factor ‘measurement time point’ (baseline vs. follow-up) and a random intercept per subject. They were fit and *p* values were calculated based on a Kenward-Roger approximation for degrees-of-freedom using the R package afex^84^. In all mixed models, discounting preference of the other (more vs. less patient than the participant) was included as a covariate. When analysing age and developmental effects on social susceptibility, i.e. social shift towards the other, own discounting preference was included as a covariate. All continuous predictors were centred on zero. Post-hoc contrasts were computed using the R package emmeans^85^. We additionally fit general additive models using a thin plate regression spline as implemented in the R package mgcv^86^ to consider non-linear relationships between age and our outcome variables of interest. As in both these models effective degrees-of freedom ~1 indicated that the models reduced the effect of age to a linear term, we report linear mixed model results. Latent change score and mediation models were fit using the package lavaan^87^ with R code provided in^49^, freely available at https://osf.io/4bpmq/files/. In all models, we used a robust estimation procedure (‘mlr’ implemented in lavaan) to account for non-normality in the data. Plots were generated using ggplot2^88^.

### Reporting summary

Further information on research design is available in the Nature Research Reporting Summary linked to this article.

## Supplementary information

Supplementary Information

Reporting Summary

## Data Availability

All behavioural, self-report and MRI-ROI data analysed during the current study are available via the Open Science Framework (https://osf.io/jpks2/). Source data are provided with this paper.

## Source data

Source Data

## Code availability

Code for computational modelling as well as the statistical analysis is available at https://osf.io/jpks2/.
