# Supplementary material for: Preference uncertainty accounts for developmental effects on susceptibility to peer influence in adolescence
Source: Nat Commun. 2021 Jun 22;12:3823. doi: 10.1038/s41467-021-23671-2 (PMC8219700; doi:10.1038/s41467-021-23671-2)
Supplement: Supplementary file 1 — Supplementary Information [file 41467_2021_23671_MOESM1_ESM.pdf]

SUPPLEMENTARY INFORMATION

Supplementary Methods

Computational Modelling

Parameter Recovery

We checked for recoverability and potential age differences therein, in order to be certain that differences in the range of discounting rate or preference uncertainty did not affect parameter recovery in younger as compared to older participants. To do this we selected from the baseline sample the 200 youngest (ages 14.1 to 16.4 years) and 200 oldest participants (ages 21.1 to 24.5 years). We then used our generative model to create pseudo-data for each group, and compared the recovery of each of the five model parameters. All parameters showed satisfactory recoverability (rs≥.51), with the parameters of key interest for the analyses reported in the main manuscript, discounting rate ($r_{younger}$=.99, r_{older}=.99), and preference uncertainty ($r_{younger}$=.81, r_{older}=.84), showing excellent recovery that was virtually identically in both age groups (see Figure S-1). In both instances, the correlation of generative and recovered values was not significantly moderated by age (all ts<.1.34, all ps>.18). As proof of concept, using a t-test to compare the impact of age group on the recovered preference uncertainty values, we reproduced the age-related significant differences we report for preference uncertainty on the empirical data, i.e. reduced preference uncertainty in the older group (t=2.94, p=.003).

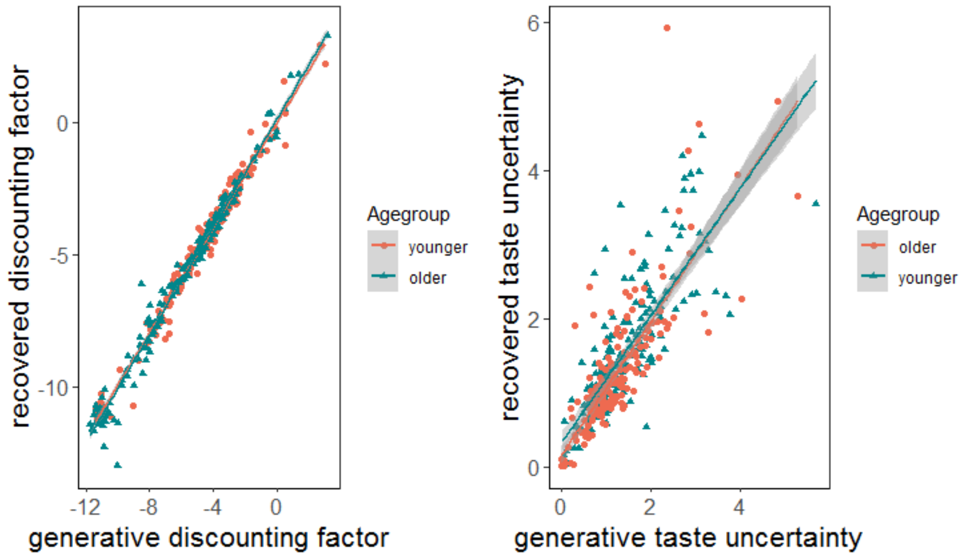

Supplementary Figure 1. Recoverability for the log discounting factor and preference uncertainty. Recoverability did not differ between younger and older participants of the sample. Error bands denote the 95% confidence interval. Source data are provided as a Source Data file.

Controlling for age-related increase in model fit

Model fit increased significantly with age (r=0.16, p<.001). To rule out a possibility that this affected age-related effects on preference uncertainty, we included the deviance measure, appropriate for sampling-method based fits as applied here, as a covariate in these analyses. Even when controlling for model fit, significant effects of age on preference uncertainty remained, both at baseline as well as at follow-up (all ps <.009).

Psychometric Measures

Perceived quality of peer relations

We used the Cambridge Friendship Questionnaire (CFQ) to assess the perceived quality of peer relations [1], a measure available as part of a Home Questionnaire Pack delivered close in time to the in-lab measurements [2]. The CFQ assesses the number, and quality of friendships via self-report (e.g. “How often do you arrange to see friends other than at school, college or work?”, “Do you feel that your friends understand you”, “Can you confide in your friends”). Higher scores signify higher satisfaction with peer

relations. This measure has been shown to predict psycho-social resilience in this sample [1].

Substance Consumption

Alcohol use was measured looking at the frequency ("Never", "Occasionally" "Often" "Every day or nearly every day") of drinking beer/cider, wine, spirits and alcopops, respectively. Cigarette use was measured by a questions about how often they smoked ("I didn't smoke" "1-10 cigarettes a day" 11-20 cigarettes a day" "More than 20 cigarettes a day"). Frequency of cannabis use in the past month was asked within one question ("Never", "Occasionally", "Often", "Daily"). To analyse the co-development of social susceptibility and substance consumption, an aggregate sum score was built.

MRI pre-processing and Region of Interest extraction

MT maps were spatially pre-processed using a standard pipeline as implemented in the hMRI toolbox. Maps were segmented using unified segmentation [3] and normalised to MNI space using Diffeomorphic Anatomical Registration using Exponentiated Lie Algebra (DARTEL[4]), followed by spatial smoothing (6mm full-width half-maximum) using tissue-weighted smoothing to preserve grey matter / white matter boundaries.

Data quality was assessed by inspection for visible motion artefacts by an expert (G.Z., compare (7)) as well as with a covariance-based measure of sample homogeneity as implemented in the CAT toolbox, identifying volumes which deviated by more than two standard deviations from the remaining sample. Removal of these scans as well as those which failed segmentation during pre-processing led to an exclusion of a total of n=55 datasets.

To additionally account for motion in the remaining datasets, head motion was approximated based on the standard deviation parameter of R2* exponential decay residuals (SDR2*), which has high sensitivity to motion-related image degradation and has been shown to be a reliable measure of across scans in the context of MPMs [5,6]. None of the participants met our SDR2* exclusion criteria due to excessive head motion (no outliers identified by using Tukey's interquartile rule, by 2.5 standard deviations above

the mean, nor by using the R function extremevalues [7]. Including SDR2* as a regressor into our analysis, did not change the reported association of mPFC myelin at T1 and the change in preference uncertainty did not change (raw beta=-3.88, standardised beta=-.13, z=-2.14, p=.03).

To add a developmentally relevant control analysis, we studied an ROI centred on the angular gyrus. This was chosen based on our previous findings [6] in the same sample, where a peak of both age-related and longitudinal change in myelin was found centred on the angular gyrus (peak region of both longitudinal and age-related myelin changes, we used an anatomically defined mask of the angular gyrus based on the probabilistic Harvard-Oxford cortical structural atlas (thresholded at 30%).

Supplementary Results

Subjects’ own (phase 1) delay discounting preferences

Subjects’ choice behaviour

Inspecting raw choice behaviour (proportion of delayed choices) in phase 1, we observed a minority of participants showing “extreme behaviour”, in the sense of showing >90% choice of either the sooner or the later option (see Supplementary Figure 2). To ensure that these participants do not drive the observed developmental effects on delay discounting, we repeated the age and longitudinal analyses on social susceptibility reported in our manuscript, while excluding participants with both forms of extreme choice behaviour.

In these analyses, age remained significantly associated with social susceptibility at baseline ($r=-.10$, $t=-2.60$, $p=.009$), and the longitudinal analysis again showed an effect of measurement time point on social susceptibility ($F(1,528.03)=6.71$, $p=.01$). In the latter analysis, the previously trend-wise significant interaction of baseline age x measurement time point was significant ($F(1,529.23)=4.65$, $p=.03$).

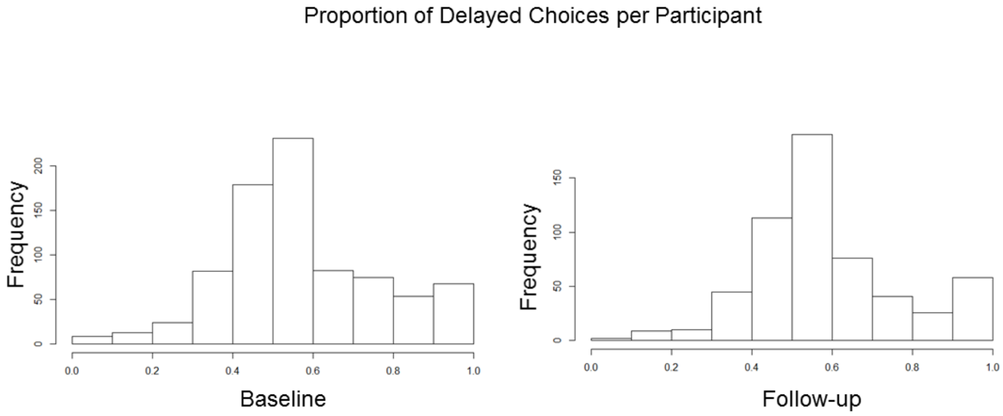

Supplementary Figure 2. Proportions of choices (Baseline / T1 and ~1.5 years Follow-up / T2) where the participant decided for the delayed option in the delay discounting task. Source data are provided as a Source Data file.

Cross sectional age effects on delay discounting

At baseline, we found a significant negative association of delay discounting, i.e. log k_{self_phase1} with age (r=-.10, p=.004, see Supplementary Figure 3), in line with previous cross-sectional observations [8-11]. This age effect was less pronounced than in some of the previous studies, possibly due to delay discounting mostly decreasing in late childhood and early adolescence, just before the age range included in our study. Note that phase 1 delay discounting preferences were included as covariates in all models predicting social susceptibility.

Longitudinal analysis

No significant longitudinal effect on delay discounting was observed in the mixed model analysis (effect of measurement time point F(1,564.82)=.71, p=.40), nor was there an interaction of baseline age with measurement time point (F(1,564.55=.26, p=.61), see S Figure 4). Thus, we consider it unlikely that the longitudinal effects observed on social susceptibility (as reported in our main manuscript) are driven by longitudinal effects on delay discounting.

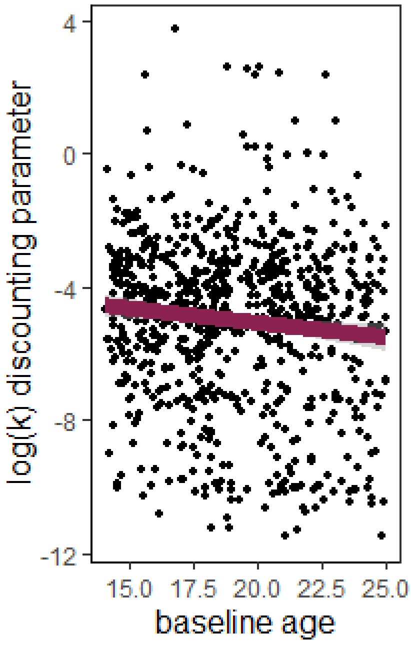

Supplementary Figure 3. At baseline, we observed a negative association of delay discounting with age, consistent with participants becoming more patient with age. The *error band denotes the 95% confidence interval. Source data are provided as a Source Data file.*

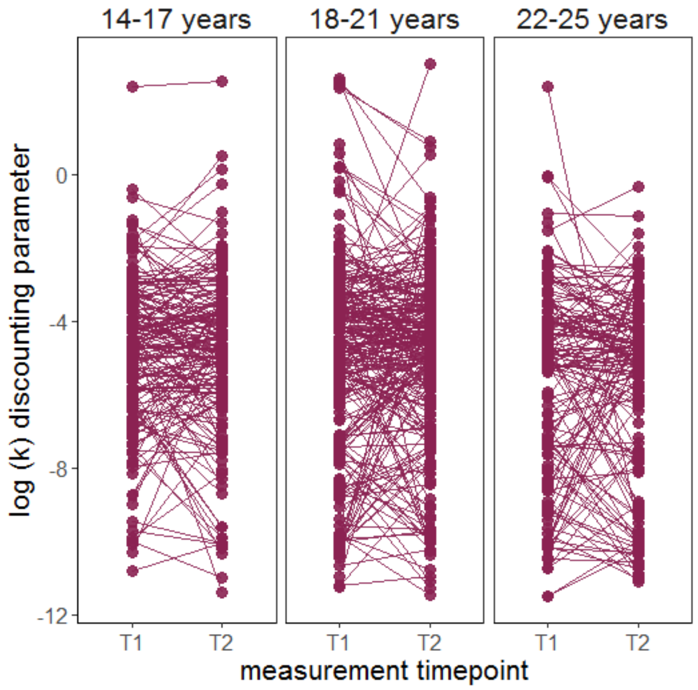

Supplementary Figure 4. Longitudinal change in delay discounting plotted as a function of baseline age. No significant longitudinal change in delay discounting over the 1.5 follow-up, nor an interaction of baseline age with longitudinal change. Note that age entered the model as a continuous regressor, here we plot 4-year-age bins ≤17 years old, >17 ≤21 years old, >21 years old) for visualization purposes alone. Source data are provided as a Source Data file.

Effect of the other's preference

Previous studies on preference shifts in adolescents, e.g. in the domain of risk-taking, have shown differential effects depending on the direction of the partner's preference (i.e. in our case, more vs. less patient than the participant). Thus, the direction of the other's preference (more vs. less patient) was included as a categorical covariate in our mixed model (see main methods), to ensure that effects on social susceptibility are independent of the other's preference. Inspecting the effect of the other's preference on social susceptibility, we find that overall participants tended to shift more towards patient others ($F(1,1125.05=30.12$, $p<.001$).

We conducted an additional control analysis to predict social susceptibility, where we included in the model a categorical regressor which indexed whether the direction of peer influence was the same or different at baseline and at follow-up. This model fit

the data less well than our reported model (ΔAIC=121, ΔBIC=16.2, p>.9). Reassuringly, inclusion of this regressor did not alter the finding of a longitudinal effect (t(564,56,=6.62, p=.01), nor did it interact significantly with the time effect (time x equal direction: t(564.56)=1.85, p=.17; age x time x equal direction: t(565.78, t=.41, p=.52), which would have indicated that longitudinal change depends on whether the direction of influence was held constant or not.

Learning about others' preferences

Trials needed to reach learning criterion.

Trials to criterion were on average m=42.61 trials (sd=15.71) for baseline, and m=42.48 (sd=15.76) for follow-up, comparable to our previous study in young adults [12]. Trials in the learning phase were not significantly associated with age neither at baseline (spearman's rho: -.06, p=.09), nor at the 1.5 years follow-up measurement (spearman's rho: .05, p=.21).

Below threshold learning performance

Learning performance was defined as the proportion of correct choices relative to all phase 2 trials. We defined a cut-off of 62% for "above-chance learning" (based on a significant binomial test (null hypothesis=random performance of 50% correct, p<.05 one-sided for "higher than chance performance"). Based on this cut-off, we identified n=40 participants for T1 and n=26 participants for T2 who showed below-threshold learning (≤5% poor learners).

To ensure our developmental results are not affected by these poor learners, we re-run the analyses on the development of i) social susceptibility and ii) preference uncertainty excluding these poor learners.

For social susceptibility, this revealed the effects of baseline age and measurement time point on social susceptibility remained significant (age: F(1,552.47)=4.058, p=.044, time: (F1,549.66)=5.18, p=.023). The previously trend-wise interaction of measurement time point and baseline age (see main results) now attained significance (F(1,550.12)=4.42, p=0.036) in this analysis.

Likewise, for preference uncertainty, as reported in the original analysis, we observed significant effects of baseline age F(1,549.94)=13.39, p<.001) and measurement time

point (F(1,544.94)=5.00, p=.026), as well as the interaction of both (F(1,545.45)=10.46, p=.001).

Reaction Times

We analysed whether there were significant developmental effects on mean reaction time (RT, Phase 1, Phase 2, Phase 3 of the task). There was no significant effect of age on RT in neither of the task phases, neither at baseline nor on follow-up (all ps>.14, all rs<.07), however, measurement time point influenced RT, with subjects becoming faster from baseline to follow-up (all Fs>26.59, all ps<.001). This is in line with a general notion of a developmental increase in processing speed, albeit more pronounced from childhood to early adolescence than in the age range we are studying [13,14].

Computational Modelling

Correlation of Preference Uncertainty and Social Susceptibility

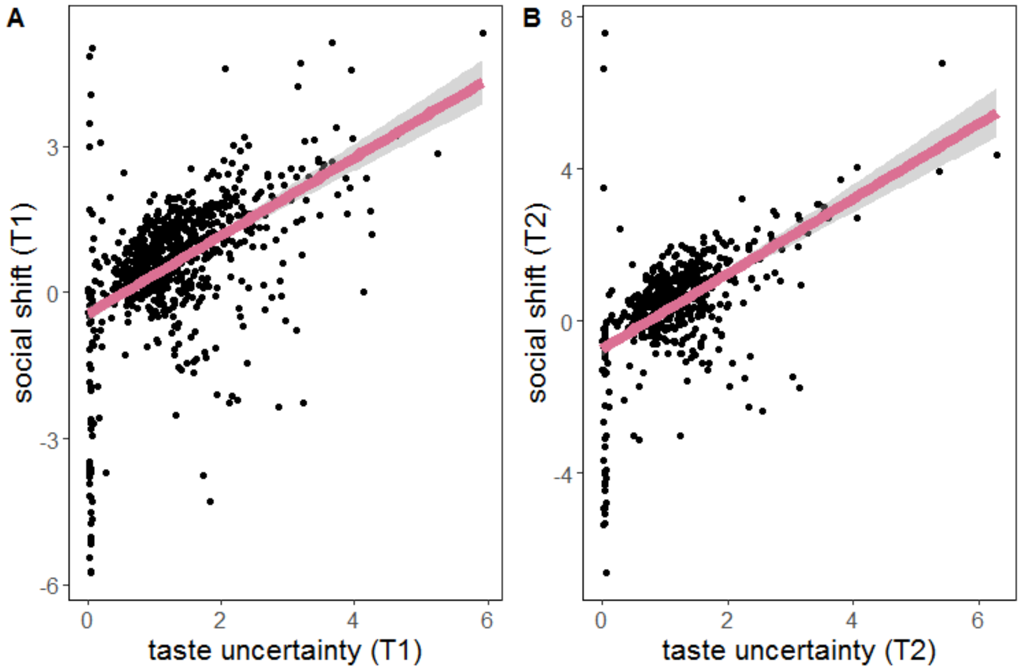

Supplementary Figure 5. Preference uncertainty and social susceptibility. Preference uncertainty significantly predicted social susceptibility at both T1 (Panel A) and T2 (Panel B), in line with an informational account of conformity[15]. Error bands denote the 95% confidence interval. Source data are provided as a Source Data file.

'Relevance of the Other' Parameter

Note that apart from preference uncertainty, which is the focus of our developmental study here, also a second parameter, namely 'relevance of the other' (see [16] and Methods for details) accounts for social shift in our computational model (all r <-.25, all t<-6.24, all p<8.4e-10). There was no significant age correlation with the 'relevance of the other' parameter (r=.006, t=0.19, df=780 p=.848). Whilst our developmental hypothesis and experimental design focussed on the preference uncertainty parameter of the model, in future research a different framing of our task could explicitly manipulate the relevance of the social influence (e.g., as a function of age group (compare, e.g. [17,18]), which might result in developmentally sensitive differences in the 'relevance of the social partner' parameter.

Control analysis: mPFC myelin marker and its relation to model-based decision making as a control psychological construct

We used the same latent change score model as described in the manuscript, but replaced the Preference Uncertainty parameter with a parameter indexing model-based decision-making in a standard sequential decision-making task [19]. We found no evidence to support a model-basedness – mPFC myelin marker coupling path (all ps>.096). Therefore, although model-basedness improves with testing session [20] we found no evidence that it relates to our neural measure of interest.

Supplementary Table 1

Bivariate latent change score model: Co-development of Social susceptibility and Perceived Quality of Peer Relationships (Cambridge Friendship Questionnaire) across the whole sample. est: unstandardized estimate, se: standard error, est (stand): standardised estimate, T1: measurement time point 1, baseline assessment, T2: measurement time point 2, follow-up assessment after ~1-5 years. Note that intercepts of change scores reported in the main manuscript are estimated in a model which sets the self-feedback path to a covariance, to estimate unconditional change scores[21]. We report p-values for two-sided tests.

Parameter	est	Se	z	p	est (stand)
Intercept Change Social Susceptibility	-.342	.444	-.772	.440	-.180
Mean Social Susceptibility at T1	.591	.049	12.038	<.001	.430
Variance Change in Social Susceptibility	3.635	.381	9.532	<.001	1
Variance Social Susceptibility at T1	1.886	.173	10.877	<.001	1
Intercept Change in Friendship Quality	.313	.137	2.284	.022	.094
Mean Friendship Quality at T1	23.104	.136	170.358	<.001	6.105
Variance Change in Friendship Quality	11.079	.836	13.245	<.001	.994
Friendship Quality at T1 Variance	14.323	.925	15.489	<.001	1
Regression Paths					
Change in Friendship Quality~ Social Susceptibility at T1	.188	.089	2.118	.034	.078
Change in Social Susceptibility ~ Friendship Quality at T1	.007	.019	.380	.704	.014
Covariance Paths					
Covariation Friendship Quality at T1 and Change in Friendship Quality	-5.575	.595	-9.371	<.001	-.443
Covariation Social Susceptibility at T1 and Change in Social Susceptibility	-1.738	.205	-8.460	<.001	-.664
Covariation Social Susceptibility at T1 and Friendship Quality at T1	-.132	.171	-.774	.439	-.025
Covariation Change in Social Susceptibility and Change in Friendship Quality	.044	.219	.838	.448	.007

Supplementary Table 2

Latent change score model: Longitudinal development of Social susceptibility and Preference uncertainty. Preference uncertainty covaries with social susceptibility at T1 and longitudinal change in preference uncertainty covaries with longitudinal change in social susceptibility. Note that intercepts of change scores reported in the main manuscript are estimated in a model which sets the self-feedback path to a covariance, to estimate unconditional change scores[21].est: unstandardised estimate, se: standard error, est (stand): standardised estimate, T1: measurement time point 1, baseline assessment, T2: measurement time point 2, follow-up assessment after ~1-5 years. We report p-values for two-sided tests.

Parameter	est	se	z	p	est (stand)
Intercept Change in Social Susceptibility	-0.176	.082	-2.157	.031	-.094
Mean Social Susceptibility at T1	.599	.060	9.982	<.001	.432
Variance Change in Social Susceptibility	3.499	.394	8.889	<.001	1.000
Variance Social Susceptibility at T1	1.926	.204	9.448	<.001	1.000
Intercept Change in Preference Uncertainty	-.106	.046	-2.317	.020	-0.100
Mean Preference Uncertainty at T1	1.277	.036	35.225	<.001	.555
Variance Change in Preference Uncertainty	1.142	.105	10.909	<.001	1.000
Variance Preference Uncertainty at T1	.675	.062	10.921	<.001	1.000
Covariation Preference Uncertainty at T1 and Change in Preference Uncertainty	-.555	.077	-7.224	<.001	-.632
Covariation Social Susceptibility at T1 and Change in Social Susceptibility	-1.605	.248	-6.462	<.001	-.618
Covariation Social Susceptibility at T1 and Preference Uncertainty at T1	.310	.045	6.852	<.001	.272
Covariation Change in Social Susceptibility and Change in Preference Uncertainty	.409	.107	3.815	<.001	.205

Supplementary Table 3

Bivariate latent change score model: Co-development of preference uncertainty and intra-cortical myelin marker in mPFC. est: unstandardised estimate, se: standard error, est (stand): standardised estimate, mPFC: medial prefrontal cortex, Myelin: Myelin Marker as estimated via quantitative magnetization transfer imaging, T1: measurement time point 1, baseline assessment, T2: measurement time point 2, follow-up assessment after ~1-5 years. We report p-values for two-sided tests.

Parameter	est	se	z	p	est (stand)
Intercept Change Preference Uncertainty	5.301	1.576	3.364	<.001	4.748
Mean Preference Uncertainty at T1	3.133	.633	4.949	<.001	3.605
Variance Change in Preference Uncertainty	.594	.119	5.010	<.001	0.477
Variance in Preference Uncertainty at T1	.726	.129	5.639	<.001	0.960
Intercept Change mPFC Myelin	.532	.054	9.914	<.001	14.774
Mean mPFC Myelin at T1	.746	.029	25.365	<.001	20.825
Variance mPFC Myelin Change	.001	.000	7.309	<.001	.593
Variance mPFC Myelin at T1	.001	.000	7.439	<.001	.900
Regression Paths					
Change mPFC Myelin ~ Mean Preference Uncertainty at T1	.004	.003	1.526	0.127	.096
Change Preference Uncertainty ~ Mean mPFC Myelin at T1	-3.888	1.815	-2.142	.032	-.125
Self-Feedback mPFC Myelin	-.632	.071	-8.894	<.001	-.630
Self-Feedback Preference Uncertainty	-.917	.071	-12.931	<.001	-.715
Covariance Paths					
Covariance Preference Uncertainty at T1 and mPFC Myelin at T1	.001	.002	.454	.650	.030
Covariance Change in Preference Uncertainty and Change in mPFC Myelin at T1	-.001	.001	-1.052	0.293	-.070
**Covariates**					
Age					
Preference Uncertainty ~ Age	-3.290	1.926	-1.708	.088	-.110
Change in Preference uncertainty ~ Age	1.982	2.146	.923	.356	.052
Myelin at T1 ~ Age	.255	.094	2.705	.007	.206
Change in Myelin ~ Age	.073	.077	.952	.341	.059
Sex (dummy-coded)					
Preference Uncertainty ~ Sex	-.043	.124	-.347	.729	-.025
Change in Preference Uncertainty ~ Sex	-.196	.114	-1.720	.085	-.088
Myelin at T1 ~ Sex	-.008	.005	-1.641	.101	-.113
Change in Myelin ~ Sex	-.009	.004	-2.228	.026	-.127
IQ					
Preference Uncertainty ~ IQ	-1.160	.467	-2.484	.013	-.150
Change in Preference Uncertainty ~ IQ	-1.327	.469	-2.831	.005	-.134
Myelin at T1 ~ IQ	.006	.022	.298	.766	.020
Change in Myelin ~ IQ	-.031	.016	-1.934	.053	-.096
Site (dummy-coded)					
Myelin at T1 ~ Site 1 at T1	-.015	.007	-2.255	.024	-.145
Myelin at T1 ~ Site 2 at T1	-.016	.008	-2.051	.040	-.156
Myelin at T2 ~ Site at T2	-.002	.005	-.375	.708	-.019

Supplementary References

1. van Harmelen, A.L., *et al.* Adolescent friendships predict later resilient functioning across psychosocial domains in a healthy community cohort. *Psychol Med* **47**, 2312-2322 (2017).
2. Kiddle, B., *et al.* Cohort profile: the NSPN 2400 Cohort: a developmental sample supporting the Wellcome Trust NeuroScience in Psychiatry Network. *International journal of epidemiology* **47**, 18-19g (2017).
3. Ashburner, J. & Friston, K.J. Unified segmentation. *Neuroimage* **26**, 839-851 (2005).
4. Ashburner, J. A fast diffeomorphic image registration algorithm. *Neuroimage* **38**, 95-113 (2007).
5. Castella, R., *et al.* Controlling motion artefact levels in MR images by suspending data acquisition during periods of head motion. *Magnetic resonance in medicine* **80**, 2415-2426 (2018).
6. Ziegler, G., *et al.* Compulsivity and impulsivity traits linked to attenuated developmental fronto-striatal myelination trajectories. *Nature Neuroscience* **22**, 992 (2019).
7. van der Loo, M. extremevalues, an R package for outlier detection in univariate data. *R package version* **2**(2010).
8. van den Bos, W., Rodriguez, C.A., Schweitzer, J.B. & McClure, S.M. Adolescent impatience decreases with increased frontostriatal connectivity. *Proc Natl Acad Sci U S A* **112**, E3765-3774 (2015).
9. Olson, E.A., Hooper, C.J., Collins, P., Luciana, M.J.P. & differences, i. Adolescents' performance on delay and probability discounting tasks: contributions of age, intelligence, executive functioning, and self-reported externalizing behavior. **43**, 1886-1897 (2007).
10. Laube, C., Suleiman, A.B., Johnson, M., Dahl, R.E. & van den Bos, W. Dissociable effects of age and testosterone on adolescent impatience. *Psychoneuroendocrinology* **80**, 162-169 (2017).
11. Achterberg, M., Peper, J.S., van Duijvenvoorde, A.C., Mandl, R.C. & Crone, E.A. Frontostriatal White Matter Integrity Predicts Development of Delay of Gratification: A Longitudinal Study. *J Neurosci* **36**, 1954-1961 (2016).
12. Garvert, M.M., Moutoussis, M., Kurth-Nelson, Z., Behrens, T.E. & Dolan, R.J. Learning-induced plasticity in medial prefrontal cortex predicts preference malleability. *Neuron* **85**, 418-428 (2015).
13. Kail, R. Developmental change in speed of processing during childhood and adolescence. *Psychological bulletin* **109**, 490 (1991).
14. Fry, A.F. & Hale, S. Processing speed, working memory, and fluid intelligence: Evidence for a developmental cascade. *Psychological science* **7**, 237-241 (1996).
15. Toelch, U. & Dolan, R.J. Informational and Normative Influences in Conformity from a Neurocomputational Perspective. *Trends Cogn Sci* **19**, 579-589 (2015).
16. Moutoussis, M., Dolan, R.J. & Dayan, P. How People Use Social Information to Find out What to Want in the Paradigmatic Case of Inter-temporal Preferences. *Plos Computational Biology* **12**, e1004965 (2016).
17. Reiter, A.M.F., Suzuki, S., O'Doherty, J.P., Li, S.C. & Eppinger, B. Risk contagion by peers affects learning and decision-making in adolescents. *J Exp Psychol Gen* **Advance online publication**(2019).
18. Knoll, L.J., Magis-Weinberg, L., Speekenbrink, M. & Blakemore, S.-J. Social influence on risk perception during adolescence. *Psychological science*, 0956797615569578 (2015).
19. Daw, N.D., Gershman, S.J., Seymour, B., Dayan, P. & Dolan, R.J. Model-based influences on humans' choices and striatal prediction errors. *Neuron* **69**, 1204-1215 (2011).
20. Vaghi, M.M., *et al.* Compulsivity is linked to reduced adolescent development of goal-directed control and frontostriatal functional connectivity. *Proceedings of the National Academy of Sciences* **117**, 25911-25922 (2020).

21. Kievit, R.A., *et al.* Developmental cognitive neuroscience using latent change score models: A tutorial and applications. *Developmental Cognitive Neuroscience* **33**, 99-117 (2018).
