# Supplementary material for: Preference uncertainty accounts for developmental effects on susceptibility to peer influence in adolescence
Source: Nat Commun. 2021 Jun 22;12:3823. doi: 10.1038/s41467-021-23671-2 (PMC8219700; doi:10.1038/s41467-021-23671-2)
Supplement: Supplementary file 2 — Reporting Summary [file 41467_2021_23671_MOESM2_ESM.pdf]

nature research

Corresponding author(s): Andrea Reiter

Last updated by author(s): Mar 18, 2021

Reporting Summary

Nature Research wishes to improve the reproducibility of the work that we publish. This form provides structure for consistency and transparency in reporting. For further information on Nature Research policies, see Authors & Referees and the Editorial Policy Checklist.

Statistics

For all statistical analyses, confirm that the following items are present in the figure legend, table legend, main text, or Methods section.

n/a	Confirmed	
☐	☒	The exact sample size (n) for each experimental group/condition, given as a discrete number and unit of measurement
☐	☒	A statement on whether measurements were taken from distinct samples or whether the same sample was measured repeatedly
☐	☒	The statistical test(s) used AND whether they are one- or two-sided<br>*Only common tests should be described solely by name; describe more complex techniques in the Methods section.*
☐	☒	A description of all covariates tested
☐	☒	A description of any assumptions or corrections, such as tests of normality and adjustment for multiple comparisons
☐	☒	A full description of the statistical parameters including central tendency (e.g. means) or other basic estimates (e.g. regression coefficient) AND variation (e.g. standard deviation) or associated estimates of uncertainty (e.g. confidence intervals)
☐	☒	For null hypothesis testing, the test statistic (e.g. F, t, r) with confidence intervals, effect sizes, degrees of freedom and P value noted<br>*Give P values as exact values whenever suitable.*
☒	☐	For Bayesian analysis, information on the choice of priors and Markov chain Monte Carlo settings
☒	☐	For hierarchical and complex designs, identification of the appropriate level for tests and full reporting of outcomes
☒	☐	Estimates of effect sizes (e.g. Cohen's d, Pearson's r), indicating how they were calculated

Our web collection on statistics for biologists contains articles on many of the points above.

Software and code

Policy information about availability of computer code

Data collection	Data were collected using MATLAB 2012a with the Cogent graphic toolbox (http://www.vislab.ucl.ac.uk/Cogent/)
Data analysis	Data were analysed using R 3.4.3 82 with R Studio Version 1.1.383 for behavioural analyses; SPM12 including DARTEL and hMRI toolbox and FSL was used for structural MRI.

For manuscripts utilizing custom algorithms or software that are central to the research but not yet described in published literature, software must be made available to editors/reviewers. We strongly encourage code deposition in a community repository (e.g. GitHub). See the Nature Research guidelines for submitting code & software for further information.

Data

Policy information about availability of data

All manuscripts must include a data availability statement. This statement should provide the following information, where applicable:

- Accession codes, unique identifiers, or web links for publicly available datasets
- A list of figures that have associated raw data
- A description of any restrictions on data availability

Behavioural, self-report and MRI data analysed during the current study are available via the Open Science Framework (https://osf.io/jpks2/).

Field-specific reporting

Please select the one below that is the best fit for your research. If you are not sure, read the appropriate sections before making your selection.

☐ Life sciences ☒ Behavioural & social sciences ☐ Ecological, evolutionary & environmental sciences

October 2018

Behavioural & social sciences study design

All studies must disclose on these points even when the disclosure is negative.

Study description	We report quantitative cross-sectional and longitudinal data using experimental task-measures, self-report questionnaires and structural MRI data in young human participants (14-24 yrs old).
Research sample	Participants were part of the larger NSPN Cohort which recruited >2000 participants in an age- sex-stratified sample, including equal numbers of males and females for the following five age groups: 14-15, 16-17, 18-19, 20-21, and 22-24.99years. For more details on the sample, please see the accompanying paper, Kiddle et al. 2018, Int J Epidemiol, for a detailed sample description.
Sampling strategy	Participants were part of the larger NSPN Cohort which recruited >2000 participants in an age- sex-stratified sample, including equal numbers of males and females for the following five age groups: 14-15, 16-17, 18-19, 20-21, and 22-24.99years. The NIHR Primary Care Research Network (PCRN) engaged 50 GP's to recruit young people using their sex-age registers by sending out invitations (including an expressions of interest form (EoI)) across Cambridgeshire and Greater London (closest proximity to universities leading the study). Schools and Further Education colleges were also engaged to distribute the EoI forms to 14 to 18-year-old participants. The NSPN recruitment team assisted GP's and schools by providing invitation to participate letters, which were forwarded to potential participant's home address that remained unknown to the NSPN investigators. Sample sizes were chosen to be substantially larger than in comparable studies demonstrating significant peer influence in adolescents on a cross-sectional level (compare, e.g. , Reiter et al., 2019, JEP General: n=86; Knoll et al., 2016, Psych Science: n=560; Chein et al. et al., 2011, Dev Science n=40).
Data collection	Behavioural data were collected using computerized tasks in the lab. A trained RA conducted the experiments. During the cognitive task performance there was no-one else present. The trained RAs were blind to the specific (e.g., age-related/developmental) hypotheses of this study, and the concept of 'dicounting preference uncertainty' was unknown to them. They were aware that the task related to impulsivity and peer influence, and knew that the participants were playing with a computer, not with a real person.<br>Self-report data were collected via paper-and-pencil questionnaires sent to the participants' home address.
Timing	Data were collected between 2012-2017
Data exclusions	No data were excluded for behavioural analyses. In the computational modeling analysis, our algorithm did not show convergence in n=2 datasets, which were thus excluded from the computational modeling analyses; MRI data were excluded after visual and quantitative inspection for quality assessment (e.g. due to excessive head motion, as described in the paper)
Non-participation	For the given task, 568 of originally 784 participants returned for the follow-up. approximately 1.5yrs later. This corresponds to a return rate of 72%, which is comparable with attrition rates of other European large-scale studies on adolescent development, such as the IMAGEN consortium (Schumann et al., 2012), reporting an average of 67.7% of longitudinal return rates over a 2-year follow-up period (O'Leary-Barrett et al., 2015). We have only anecdotal insight into why subjects did not return for the follow-up, though we suspect reasons are common factors in this age group such as geographical relocation and embarking on new educational courses/employment.
Randomization	Participants were not allocated to experimental groups.

Reporting for specific materials, systems and methods

We require information from authors about some types of materials, experimental systems and methods used in many studies. Here, indicate whether each material, system or method listed is relevant to your study. If you are not sure if a list item applies to your research, read the appropriate section before selecting a response.

Materials & experimental systems

n/a	Involved in the study	
☒	☐	Antibodies
☒	☐	Eukaryotic cell lines
☒	☐	Palaeontology
☒	☐	Animals and other organisms
☐	☒	Human research participants
☒	☐	Clinical data

Methods

n/a	Involved in the study	
☒	☐	ChIP-seq
☒	☐	Flow cytometry
☐	☒	MRI-based neuroimaging

Human research participants

Policy information about studies involving human research participants

Population characteristics	Data for this task were available from n=784 (401 female) participants for baseline. Participants were 515 14.10-24.99 years old (mean=19.05, sd=2.96) at baseline. N=569 (284 female) participants returned for a second assessment approximately 1.5 years later. Mean age at follow-up was 20.29 years (range: 15.11- 26.48 years, sd=2.97) while mean time between first and second assessment was 1.48 years (range: 0.98-2.62 years, sd=0.30). For more details on the population, please see the accompanying paper, Kiddle et al. 2018, Int J Epidemiol, for a detailed description.

Recruitment	Recruitment is described in detail in the accompanying paper describing the cohort study, Kiddle et al. 2018, Int J Epidemiol. In brief, participants were part of the larger NSPN Cohort which recruited >2000 participants in an age- sex-stratified sample, including equal numbers of males and females for the following five age groups: 14-15, 16-17, 18-19, 20-21, and 22-24.99years. The NIHR Primary Care Research Network (PCRN) engaged 50 GP's to recruit young people using their sex-age registers by sending out invitations (including an expressions of interest form (EoI)) across Cambridgeshire and Greater London (closest proximity to universities leading the study). Schools and Further Education colleges were also engaged to distribute the EoI forms to 14 to 18-year-old participants. The NSPN recruitment team assisted GP's and schools by providing invitation to participate letters, which were forwarded to potential participant's home address that remained unknown to the NSPN investigators. Purposive advertisement was also used during recruitment; invitation letters with EoI were sent to those who responded to advertisements that met the age criteria. If an individual wanted to participate they informed NSPN recruitment team over the phone/sent in completed EoI form. The key self-selection bias that we identified was a differential self-selection into the 'cognitive cohort' and the 'scanning cohort' on the basis of socio-economic status and an interaction of self-reported gender and general cognitive ability, as measured by IQ. Namely, we observed that more male participants of higher IQ self-selected for the cognitive cohort; and that fewer young people of low socioeconomic status, compared to England as a whole, selected themselves for both the cognitive and MRI cohorts. Self-selection biases in terms of socio-economic status and IQ are likely to be very common in published studies in Developmental Psychology/Neuroscience.<br>We also point out that the study samples focused on the healthy population by construction. A more detailed analysis of demographic differences between the baseline population, based on census data, and the analysed samples can be found in (see also Figure S2 of the Supplement of) Ziegler at al, 2020, Human Brain Mapping. We cannot rule out the possibility that these self-selection and related biases limit the generalizability of our results, as we note in our discussion section.
Ethics oversight	All participants provided written informed consent, if a particpant was aged <16, consent was also obtained from their legal guardian. The Cambridge Central Research Ethics Committee approved the study (12/EE/0250).

Note that full information on the approval of the study protocol must also be provided in the manuscript.

Magnetic resonance imaging

Experimental design

Design type	Magnetisation transfer weighted imaging (structural)
Design specifications	N/A
Behavioral performance measures	N/A

Acquisition

Imaging type(s)	Structural
Field strength	3T
Sequence & imaging parameters	Multi-echo FLASH magnetization transfer weighted contrast at 1mm isotropic resolution (TR: 23.7, α =6°, 176 sagittal slices, FOV=256 mm × 240 mm, matrix = 256 × 240 × 176).
Area of acquisition	whole-brain

Diffusion MRI ☐ Used ☒ Not used

Preprocessing

Preprocessing software	Quantitative magnetization transfer saturation (MT) maps were derived using biophysical models with the hMRI toolbox (www.hmri.info) for SPM 12 (Wellcome Centre for Human Neuroimaging, London, UK, http://www.fil.ion.ucl.ac.uk/spm).<br>MT maps were spatially pre-processed using the hMRI toolbox. Maps were segmented using unified segmentation and normalised to MNI space using Diffeomorphic Anatomical Registration using Exponentiated Lie Algebra (DARTEL), followed by spatial smoothing (6mm full width half-maximum) using tissue-weighted smoothing to preserve grey matter / white matter boundaries.
Normalization	affine, nonlinear (DARTEL)
Normalization template	MNI
Noise and artifact removal	First, scans were manually inspected by an expert for motion artefacts or segmentation failure, which led to the a-priori exclusion of a total of n=55 datasets. To additionally account for motion in the remaining datasets, head motion was approximated based on the standard deviation parameter of R2* exponential decay residuals (SDR2*), which has high sensitivity to motion-related image degradation and has been shown to be a reliable measure of across scans in the context of MPMs. Including SDR2* as a regressor into our analysis did not change the results.
Volume censoring	N/A

Statistical modeling & inference

Model type and settings	Mean MT values from within a mask of medial prefrontal cortex (mPFC) were extracted from each map and entered into a latent Change Score Model (Structural Equation Model)
Effect(s) tested	Bivariate latent change score model (path model) which tested for the development of taste uncertainty and of mPFC MT, correlated change in both measures and paths in which baseline values of one of the measures predicts development of the other

Specify type of analysis: ☐ Whole brain ☒ ROI-based ☐ Both

Anatomical location(s): mPFC based on the probabilistic Harvard-Oxford cortical structural atlas

Statistic type for inference (See Eklund et al. 2016)	Mean MT values from within this mask region were extracted from each map and entered into a latent Change Score Model (Structural Equation Model)
Correction	Mean MT values from within this mask region were extracted from each map and entered into a latent Change Score Model (Structural Equation Model)

Models & analysis

n/a	Involved in the study
☒	☐ Functional and/or effective connectivity
☒	☐ Graph analysis
☒	☐ Multivariate modeling or predictive analysis
